# Tissue-Specific Bioactive Metabolites and Antioxidant Activity in Minicitrus (*Fortunella hindsii*) and Responses to a Fruit-Associated *Weissella* Strain

**DOI:** 10.3390/antiox15081037

**Published:** 2026-08-20

**Authors:** Han Yang, Manxi Wu, Xinlin Huang, Hujing Cao, Jinping Cao, Chongde Sun, Yue Wang

**Affiliations:** 1 Laboratory of Fruit Quality Biology/The State Agriculture Ministry Laboratory of Horticultural Plant Growth, Development and Quality Improvement, Fruit Science Institute, College of Agriculture and Biotechnology, Zhejiang University, Hangzhou 310058, China; 22116154@zju.edu.cn (H.Y.); mxwu@zju.edu.cn (M.W.); 22516204@zju.edu.cn (X.H.); 22516127@zju.edu.cn (H.C.); caojinpingabc@126.com (J.C.); adesun2006@zju.edu.cn (C.S.); 2 Center for Balance Architecture, Zhejiang University, Hangzhou 310058, China; 3 The Architectural Design & Research Institute of Zhejiang University Co., Ltd., Hangzhou 310058, China

**Keywords:** minicitrus, antioxidant activity, endophytic bacteria, microbiome, *Weissella*

## Abstract

Minicitrus (*Fortunella hindsii*) is a wild kumquat with potential nutritional and medicinal value, but its tissue-specific bioactive metabolites, antioxidant activity, and endophytes remain poorly characterized. Here, we integrated LC-MS profiling, HPLC quantification, chemical and cellular antioxidant assays, 16S rRNA gene sequencing, bacterial isolation, and treatment experiments across five tissues. Among all the metabolites determined by HPLC, 13 were quantified using authentic standards and 24 were semi-quantified as linarin equivalents. Leaves, stems, and fruits accumulated abundant flavone glycosides, with phloretin-3′,5′-di-C-glucoside reaching 1823.19 ± 278.71 μg/g FW in leaves and 847.11 ± 27.28 μg/g FW in fruits, while roots and seeds showed distinct coumarin- and furanocoumarin-rich profiles, respectively. Leaf extracts showed the strongest chemical and cellular antioxidant activities, followed by fruit extracts. Isoorientin 2″-*O*-rhamnoside and diosmin exhibited strong chemical antioxidant capacity, and several flavone glycosides showed protective effects in cellular assays. Endophytic bacterial communities differed markedly among tissues, with fruit harboring a distinct community dominated by *Weissella* and *Pantoea*, which accounted for 65.64% and 19.13% of the relative abundance, respectively. Among two culturable fruit-associated isolates, *Weissella* sp. SJG-1 treatment was associated with 32.92–104.45% increases in six HPLC-quantified metabolites and higher antioxidant activity of fruit extracts. These findings highlight minicitrus as a source of bioactive metabolites and suggest a potential association between fruit-associated bacteria and fruit functional properties.

## 1. Introduction

Citrus fruits (family Rutaceae) are widely recognized as rich sources of specialized metabolites with nutritional, sensory, and health-promoting relevance. Among these compounds, flavonoids, phenolic acids, coumarins, and related polyphenols contribute substantially to the antioxidant and other bioactive properties of citrus-derived foods and medicinal materials [1,2]. Kumquats (*Fortunella* spp.), in particular, are eaten whole (peel and pulp) and have been used in traditional medicine, reflecting their rich phytochemical content [2]. Minicitrus (*Fortunella hindsii*), a wild kumquat variety, is widely distributed in the low-altitude mountainous areas of South China. Its fruit is small, intensely sour and pungent, and has traditionally been preserved or processed into pickles, herbal teas, and seasonings in some southern regions of China [3]. Beyond its local food uses, recent phytochemical and pharmacological evidence has suggested that the fruit contains multiple bioactive constituents and exhibits potential medicinal activities, supporting its value as an edible-medicinal citrus resource within the broader concept of food–medicine homology [4]. Among these constituents, polyphenols such as flavonoids, phenolic acids, and coumarins are particularly important because they contribute substantially to the antioxidant capacity of citrus and have been associated with anti-inflammatory, cardioprotective, and anticancer effects [5]. Although the roots and leaves of minicitrus have also been used in folk medicine, the fruit is of particular interest because it represents the most directly consumable and application-oriented organ. However, compared with cultivated citrus fruits, the tissue-specific distribution of bioactive metabolites in minicitrus remains insufficiently characterized. In particular, it is still unclear which organs accumulate the major bioactive constituents and which compounds are most closely associated with the functional activity of different tissues.

In addition to host genotype and tissue identity, plant-associated microorganisms can influence plant physiology and metabolism through complex biochemical and metabolic interactions with their hosts [6,7]. Experimental studies further indicate that individual endophytic bacteria can alter plant phenotypes and specialized metabolite accumulation. However, microbiome research in citrus has focused predominantly on microbial community structure, nutrient acquisition, plant growth, and disease-related functions [8], whereas the relationship between fruit-associated endophytes and fruit phytochemical and functional quality remains poorly understood. Moreover, sequencing-based microbiome–metabolite associations alone cannot establish the functional contribution of individual bacterial taxa. Studies combining community profiling with the isolation and experimental evaluation of candidate strains have therefore become important for moving from microbiome description toward functional validation [9]. In this context, integrating metabolite and antioxidant profiling across minicitrus tissues with fruit microbiome characterization and subsequent evaluation of culturable fruit-associated bacteria provides an opportunity to identify candidate microbial factors associated with changes in fruit bioactive metabolites and functional activity.

In this study, we integrated metabolite profiling, antioxidant evaluation, and endophytic bacterial community analysis across five minicitrus tissues to characterize their phytochemical and functional diversity. We further isolated representative bacteria from fruit and evaluated whether bacterial treatment was associated with changes in fruit peel metabolites and antioxidant activity. Through this approach, we aimed to identify bioactive tissues and compounds in minicitrus, characterize its tissue-associated endophytic microbiota, and obtain preliminary strain-level evidence for the potential contribution of fruit-associated bacteria to phytochemical and functional quality.

## 2. Materials and Methods

**Figure 1:**
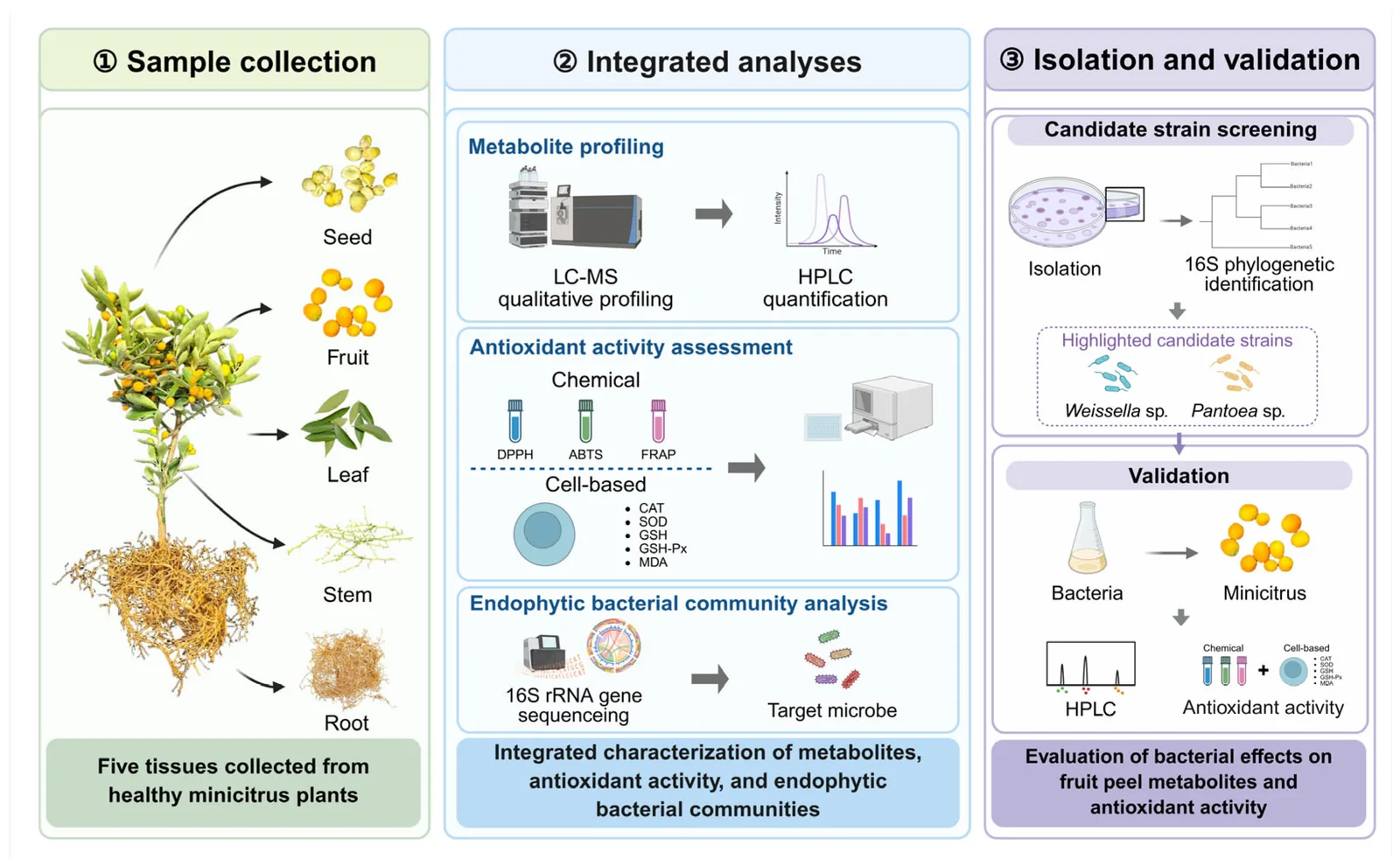
Schematic summary of the study design and main findings.

The overall study design and experimental workflow are summarized in the schematic overview (Figure 1). The study consisted of tissue-level metabolite, antioxidant, and endophytic bacterial community analyses, followed by the isolation and functional evaluation of representative fruit-associated bacterial strains.

### 2.1. Plant Materials and Sample Preparation

On 11 January 2025, twelve minicitrus plants were collected from a germplasm orchard in Longyan, Fujian Province, China. The selected plants were healthy, free of visible pests and diseases, uniform in height (35–50 cm), and each plant bore fully mature fruits. All plant materials were transported to the laboratory within 24 h after collection. The twelve plants were randomly assigned to four independent biological replicates, with each replicate consisting of three plants. For each tissue type, samples collected from the three plants within the same biological replicate were pooled, resulting in four biological replicates for subsequent analyses. The plants were washed thoroughly with tap water to remove soil and dust and then rinsed twice with sterile distilled water. Each plant was subsequently dissected into five tissues: root, stem, leaf, fruit (without seed), and seed. To eliminate microorganisms present on tissue surfaces, the samples were surface-sterilized by immersion in 1% sodium hypochlorite solution for 5 min, followed by 70% ethanol for 2 min, and finally rinsed twice with sterile distilled water [10]. To verify the effectiveness of surface sterilization, an aliquot of the final rinse water from each biological replicate was spread onto TSA agar plates and incubated under the same conditions used for bacterial cultivation. The absence of bacterial colony growth was considered confirmation of successful surface sterilization. After sterilization, tissues intended for metabolite and microbiome analyses were immediately frozen in liquid nitrogen and stored at −80 °C until further analysis.

### 2.2. Chemicals and Standards

Analytical standards used for HPLC quantification, including gentiopicrin (Gen), 7-amino-4-methylcoumarin (AMC), rhoifolin (Rho), isopimpinellin (Iso-Pim), vicenin-2 (Vic-2), xanthotoxol (Xan), auraptene (Aur), linarin (Lin), vitexin-2″-*O*-rhamnoside (Vit-Rha), diosmin (Dio), methoxsalen (MeO-xal), Isoorientin 2″-*O*-rhamnoside (Iso-Ori-2″Rha), and bergaptol (Ber), were purchased from Shanghai Yuanye Bio-Technology Co., Ltd. (Shanghai, China). Commercial assay kits for catalase (CAT), superoxide dismutase (SOD), glutathione peroxidase (GSH-Px), reduced glutathione (GSH), and malondialdehyde (MDA) were obtained from Beyotime Biotechnology (Shanghai, China). All other chemicals and solvents were of analytical or HPLC grade unless otherwise specified.

### 2.3. Extraction of Minicitrus

For extraction, the method was adapted from previously published protocols with minor modifications [11]. The five tissue types (root, stem, leaf, fruit, and seed) were first ground into a fine powder using a tissue grinder. A fresh sample of 0.1 g was weighed and extracted with 1 mL of 70% (*v*/*v*) methanol under ultrasonic-assisted conditions at 25 °C for 30 min. After centrifugation at 12,000 rpm for 20 min, the supernatant was collected. The pellet was re-extracted with another 1 mL of 80% ethanol using the same procedure. Both supernatants were combined, resulting in a final material-to-solvent ratio of 1:20 (*w*/*v*), and stored at 4 °C for further analysis.

### 2.4. Qualitative Analysis by UPLC-TripleTOF-MS/MS

The qualitative chemical profiling of minicitrus extracts was performed using a UPLC-TripleTOF^®^ 5600 mass spectrometry system (AB Sciex, Marlborough, MA, USA). Chromatographic separation was carried out on a Waters ACQUITY HSS T3 column (1.8 μm, 2.1 mm × 150 mm) with a flow rate of 0.3 mL/min. The mobile phase consisted of solvent A (0.1% formic acid with 2 mM ammonium formate in water) and solvent B (0.1% formic acid in acetonitrile). The gradient elution was programmed as follows: 0 min, 5% B; 25 min, 40% B; 35 min, 95% B; 40 min, 95% B; 41 min, 5% B. The column temperature was maintained at 30 °C, and the injection volume was 3 μL. Detection was monitored at wavelengths of 254 nm and 320 nm.

Mass spectrometric detection was performed in positive electrospray ionization (ESI+) and negative electrospray ionization (ESI-) mode with a full scan range of *m*/*z* 100–1000. The ion source parameters were set as follows: nebulizer gas (GS1) and auxiliary gas (GS2) at 55 psi, curtain gas (CUR) at 35 psi, and ion spray voltage (IS) at 5.5 kV. The declustering potential (DP) and collision energy (CE) were set at 80 V and 40 ± 20 eV, respectively. Data acquisition and compound identification were performed using PeakView software 1.2 (AB Sciex, Marlborough, MA, USA).

### 2.5. Quantitative Analysis by HPLC

The chemical constituents of minicitrus extracts were quantified using a Waters 2695 high-performance liquid chromatography system equipped with a Waters 2998 photodiode array (PDA) detector (Waters, Milford, MA, USA). Chromatographic separation was performed on a SunFire C18 column (4.6 mm × 250 mm, 5 μm; Waters, USA). The mobile phase consisted of solvent A (0.1% formic acid and 2 mM ammonium formate in water) and solvent B (0.1% formic acid in acetonitrile). The gradient program was as follows: 0 min, 5% B; 25 min, 40% B; 35 min, 95% B; 40 min, 95% B; and 41 min, 5% B. The flow rate was maintained at 1.0 mL/min, the column temperature was set at 30 °C, and the injection volume was 3 μL. Chromatograms were monitored at 254 and 320 nm.

For compounds with commercially available authentic standards, quantification was performed using compound-specific external calibration curves. Eight concentration levels were prepared for each standard, and the corresponding chromatographic peak areas were recorded. Calibration curves were constructed by linear regression using standard concentration as the independent variable and peak area as the dependent variable. The contents of individual compounds were subsequently calculated from their respective calibration equations. For compounds for which authentic standards were unavailable, semi-quantitative analysis was performed using the calibration curve of linarin, and their contents were expressed as linarin equivalents. Four independent biological replicates were analyzed for each tissue, and the quantitative results are expressed as mean ± standard deviation.

### 2.6. 16S rRNA Gene Sequencing of Bacterial Endophytes

To investigate the endophytic bacterial community composition in the five tissues of minicitrus, 16S rRNA gene amplicon sequencing was performed. Total genomic DNA was extracted from each sample using the E.Z.N.A.^®^ Soil DNA Kit (Omega Bio-Tek, Norcross, GA, USA) following the manufacturer’s instructions. The V5-V7 hypervariable regions of the 16S rRNA gene were amplified using the extracted DNA as template with the forward primer 799F (5′-AACMGGATTAGATACCCKG-3′) and reverse primer 1193R (5′-ACGTCATCCCCACCTTCC-3′) [12], both carrying unique barcode sequences. PCR amplification was conducted in a 20 μL reaction mixture containing 10 μL of 2× Pro Taq buffer, 0.8 μL each of forward and reverse primers (5 μM), approximately 10 ng of template DNA, and nuclease-free water to volume. The thermal cycling conditions were as follows: initial denaturation at 95 °C for 3 min; 27 cycles of denaturation at 95 °C for 30 s, annealing at 55 °C for 30 s, and extension at 72 °C for 45 s; followed by a final extension at 72 °C for 10 min, and holding at 4 °C. Amplification was carried out on an ABI GeneAmp^®^ 9700 thermal cycler (Applied Biosystems, Waltham, MA, USA). The PCR products from the same sample were pooled and purified from a 2% agarose gel using the AxyPrep DNA Gel Extraction Kit (Axygen Biosciences, Union City, CA, USA). The purified products were checked on 2% agarose gel and quantified using a Quantus™ Fluorometer (Promega, Madison, WI, USA).

Purified amplicons were pooled in equimolar amounts and subjected to paired-end sequencing on an Illumina MiSeq platform (Illumina, San Diego, CA, USA) by Shanghai Majorbio Bio-Pharm Technology Co., Ltd. (Shanghai, China). Raw paired-end reads were demultiplexed and quality-filtered using fastp v0.19.6. Low-quality bases were trimmed using a 50-bp sliding window when the average quality score fell below 20, and reads shorter than 50 bp after trimming or containing ambiguous bases were discarded. Paired-end reads were merged using FLASH v1.2.11, requiring a minimum overlap of 10 bp and allowing a maximum mismatch ratio of 0.2 within the overlapping region. Samples were distinguished according to their barcode and primer sequences, with no barcode mismatches and up to two primer mismatches permitted.

The quality-filtered sequences were subsequently processed using the DADA2 plugin implemented in QIIME 2 for sequence denoising, dereplication, chimera removal, and inference of amplicon sequence variants (ASVs). Taxonomic classification of representative ASV sequences was performed using the naïve Bayes classifier implemented in QIIME 2 against the SILVA 16S rRNA gene reference database (version 138). Sequences assigned to chloroplasts or mitochondria were removed before downstream analyses.

### 2.7. Chemical Antioxidant Activity Assays

The in vitro antioxidant capacities of the extracts and commercially available reference compounds were evaluated using DPPH, ABTS, and FRAP assays.

For the DPPH assay, 2 μL of diluted sample solution was mixed with 198 μL of freshly prepared DPPH solution (60 μM). The mixture was incubated in the dark at 25 °C for 2 h, after which the absorbance was measured at 517 nm using a microplate reader (Synergy H1, BioTek, Winooski, VT, USA). For the ABTS assay, the ABTS radical cation solution was prepared by mixing equal volumes of ABTS solution (7 mM) and potassium persulfate solution (2.6 mM), followed by incubation in the dark at room temperature for 12 h. Before use, the resulting solution was diluted approximately 28-fold to obtain an absorbance of approximately 0.63 at 734 nm. Subsequently, 10 μL of diluted sample solution was mixed with 200 μL of ABTS working solution and incubated in the dark at room temperature for 5 min. The absorbance was then measured at 734 nm using a microplate reader. For the FRAP assay, the FRAP working solution was freshly prepared by mixing 100 mL of sodium acetate buffer (300 mM, pH 3.6), 10 mL of TPTZ solution (10 mM), and 10 mL of FeCl_3_ solution (20 mM). Then, 20 μL of diluted sample solution was mixed with 180 μL of FRAP working solution and incubated in the dark at room temperature for 5 min. The absorbance was measured at 593 nm using a microplate reader.

### 2.8. Assessment of Cell Viability Using the CCK-8 Assay

The human normal liver cell line L02 was obtained from COBIOER Bioscience Co., Ltd. (Nanjing, China). Cells were cultured in RPMI-1640 medium supplemented with 10% fetal bovine serum and maintained at 37 °C in a humidified atmosphere containing 5% CO_2_. Cells in the logarithmic growth phase were used for subsequent experiments.

For the CCK-8 assay, L02 cells were seeded into 96-well plates at 5 × 10^4^ cells per well and cultured for 24 h. The cells were then treated with tissue extracts or individual compounds at 12.5, 25, 50, 100, and 200 μg/mL for 48 h. The final DMSO concentration was maintained below 0.5%. After treatment, the medium was replaced with 100 μL of serum-free RPMI-1640 medium containing 10% CCK-8 reagent. Following incubation for 1 h at 37 °C, absorbance was measured at 450 and 620 nm using a microplate reader. Cell viability was calculated relative to the untreated control, and concentrations maintaining > 90% cell viability were considered non-cytotoxic and selected for subsequent cellular antioxidant assays.

For establishment of the oxidative stress model, L02 cells were seeded into 6-well plates at 1 × 10^6^ cells per well and cultured for 24 h. Cells were pretreated with the selected non-cytotoxic concentrations of tissue extracts or individual compounds for 24 h, after which lipopolysaccharide (LPS, from *Escherichia coli*) was added at a final concentration of 0.1 μg/mL for an additional 12 h. The final DMSO concentration during treatment was maintained below 0.1%. For the tissue extract and standard-compound experiments, the groups included an untreated control group (CK), an LPS-treated model group (Model), five tissue extract groups, and 13 individual standard-compound groups. For the bacteria-treated fruit experiment, the groups included an untreated control, an LPS-treated model group, a group pretreated with extract from sterile-water-treated fruit peel, and a group pretreated with extract from *Weissella* sp. SJG-1-treated fruit peel. Each treatment was performed in triplicate.

### 2.9. Antioxidant Enzyme Activity Assay

Based on the CCK-8 results, 25 μg/mL was selected for subsequent cellular antioxidant assays. After treatment, cells were collected by scraping, washed twice with ice-cold PBS, and lysed in NP40 lysis buffer supplemented with protease and phosphatase inhibitors on ice. The cell lysates were centrifuged at 12,000 rpm for 15 min at 4 °C, and the supernatants were collected for subsequent biochemical analyses. Catalase (CAT) activity was determined using a Catalase Assay Kit (Beyotime, Shanghai, China, S0051), superoxide dismutase (SOD) activity using a Total Superoxide Dismutase Assay Kit (Beyotime, Shanghai, China, S0101), glutathione peroxidase (GSH-Px) activity using a Glutathione Peroxidase Assay Kit (Beyotime, Shanghai, China, S0056), reduced glutathione (GSH) content using a Reduced Glutathione Assay Kit (Beyotime, Shanghai, China, S0053), and malondialdehyde (MDA) content using a Lipid Peroxidation MDA Assay Kit (Beyotime, Shanghai, China, S0131S). All assays were performed according to the manufacturer’s instructions.

The total protein concentration of each cell lysate was determined using the bicinchoninic acid (BCA) protein assay with bovine serum albumin as the standard. All antioxidant enzyme activities and metabolite contents were normalized to the protein concentration of the corresponding sample. Each treatment was analyzed in triplicate.

### 2.10. Isolation and Identification of Minicitrus Fruit Endophytic Bacteria

Mature minicitrus fruits were used for the isolation of culturable endophytic bacteria. The biological replicate and sample-pooling design followed that described in Section 2.1. After surface sterilization and confirmation of sterilization efficiency using the final-rinse plating method described above, fruit tissues were aseptically excised, homogenized in sterile distilled water at a ratio of 1:20 (*w*/*v*), and filtered to remove large tissue debris. Aliquots of 100 μL of each filtrate were spread onto TSA, 10% TSA, NA, and LB agar plates, with three replicate plates prepared for each medium. The plates were incubated at 28 °C and 70% relative humidity for 24–48 h. Colonies with distinct morphological characteristics, including colony shape, size, color, margin, and opacity, were selected and repeatedly streaked on the corresponding agar medium until pure cultures were obtained. Purified isolates were inoculated into the corresponding liquid media and cultured at 28 °C with shaking at 200 rpm. For long-term storage, bacterial cultures were mixed with sterile 50% glycerol at a 1:1 ratio to obtain a final glycerol concentration of 25% and stored at −80 °C until further use. Before molecular identification, glycerol stocks were revived on the corresponding solid media, and single colonies were transferred into liquid media and cultured with shaking until visible turbidity developed. Bacterial cells were then collected in sterile 1.5 mL centrifuge tubes for subsequent analysis. The 16S rRNA gene of each purified isolate was amplified and sequenced by Tsingke Biotechnology Co., Ltd., Beijing, China. The obtained sequences were queried against the NCBI nucleotide database using BLAST (https://www.ncbi.nlm.nih.gov/), and taxonomic identities were assigned based on sequence similarity to the best-matching reference sequences.

### 2.11. Bacterial Inoculation Treatment of Fruit

The selected endophytic bacterial isolates were cultured in the appropriate liquid media under suitable growth conditions. Bacterial cells were collected, resuspended in sterile distilled water, and adjusted to an OD_600_ of 0.8 before treatment. Surface-sterilized mature minicitrus fruits with seeds removed were randomly assigned to the treatment groups. Fifteen fruits were treated together and pooled as one biological replicate, and four independent biological replicates were prepared for each treatment, corresponding to a total of 60 fruits per treatment. The bacterial suspension was uniformly sprayed onto the fruit surface under aseptic conditions, while sterile distilled water was applied to the control fruits using the same procedure. After natural air-drying, all fruits were incubated under sterile conditions at 25 °C for 24 h. At the end of the treatment, fruits within each biological replicate were pooled, immediately frozen in liquid nitrogen, and used for subsequent extraction, HPLC analysis, and antioxidant assays as described above.

### 2.12. Statistical Analysis

Statistical analyses and part of the data visualization were performed using GraphPad Prism 10.2.3 (GraphPad Software, Boston, MA, USA). Data are presented as mean ± standard deviation (SD). Comparisons between two independent groups were performed using two-sided unpaired Student’s *t*-tests. Comparisons among multiple groups were performed using one-way analysis of variance (ANOVA) followed by Tukey’s multiple comparisons test. For microbiome analyses, differences in alpha-diversity indices among tissue groups were assessed using the Kruskal–Wallis rank-sum test, and differences in bacterial community structure were evaluated using analysis of similarities (ANOSIM). The number of independent biological replicates (*n*) is indicated in the corresponding figure legends. A two-sided *p* value < 0.05 was considered statistically significant.

## 3. Results

### 3.1. Metabolite Distribution Among Different Tissues of Minicitrus

**Figure 2:**
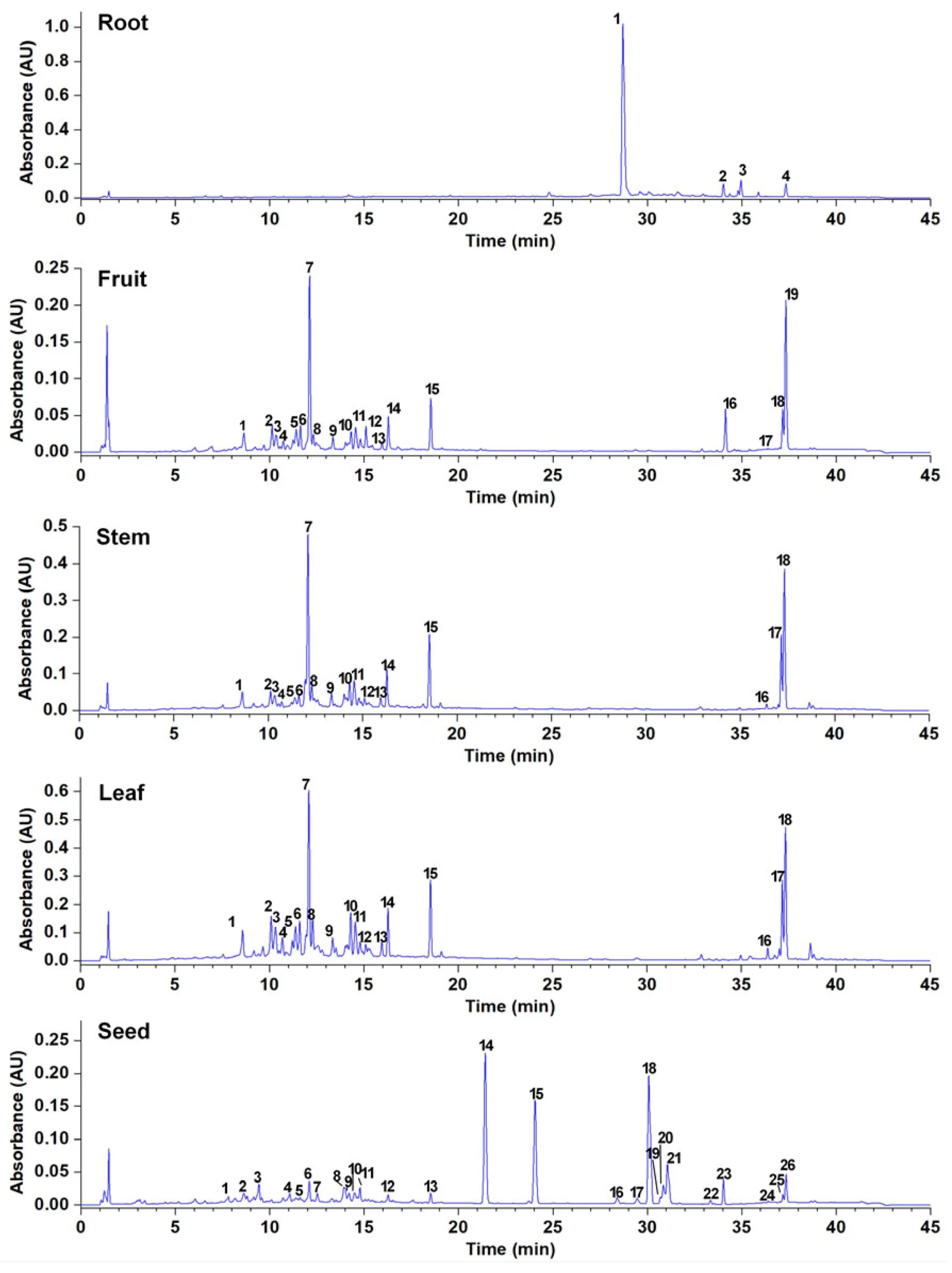
LC-MS chromatograms of root, fruit, stem, leaf, and seed tissues of minicitrus. (Root) 1. Xanthyletin; 2. Auraptene; 3. Linolenic acid; 4. 4*α*-formyl-4*β*-methyl-5*α*-8-cholesten-3*β*-ol. (Fruit) 1. Vicenin-2; 2. Isoorientin 7-*O*-rhamnoside; 3. Isoorientin 2″-*O*-rhamnoside; 4. Hedyotoside; 5. Vitexin-2″-*O*-rhamnoside; 6. Isovitexin-2″-*O*-rhamnoside; 7. Phloretin-3′,5′-di-*C*-glucoside; 8. Diosmin; 9. Limocitrin; 10. Deoxynivalenol-3-glucuronide; 11. Swertisin-2″-*O*-rhamnoside; 12. Linariifolioside; 13. Rhoifolin; 14. Isowertin-2″-*O*-rhamnoside; 15. Linarin; 16. Auraptene; 17. 1-*O*-benzoyl-17-defurano-17-(2-buten-4-olide-2-yl)salannic acid methyl ester; 18. Pyrophaeophorbide A; 19. 4*α*-formyl-4*β*-methyl-5*α*-8-cholesten-3*β*-ol. (Stem) 1. Vicenin-2; 2. Isoorientin 7-*O*-rhamnoside; 3. Isoorientin 2″-*O*-rhamnoside; 4. Hedyotoside; 5. Vitexin-2″-*O*-rhamnoside; 6. Isovitexin-2″-*O*-rhamnoside; 7. Phloretin-3′,5′-di-*C*-glucoside; 8. Diosmin; 9. Limocitrin; 10. Deoxynivalenol-3-glucuronide; 11. Swertisin-2″-*O*-rhamnoside; 12. Linariifolioside; 13. Rhoifolin; 14. Isowertin-2″-*O*-rhamnoside; 15. Linarin; 16. 1-*O*-benzoyl-17-defurano-17-(2-buten-4-olide-2-yl)salannic acid methyl ester; 17. Pyrophaeophorbide A; 18. 4*α*-formyl-4*β*-methyl-5*α*-8-cholesten-3*β*-ol. (Leaf) 1. Vicenin-2; 2. Isoorientin 7-*O*-rhamnoside; 3. Isoorientin 2″-*O*-rhamnoside; 4. Hedyotoside; 5. Vitexin-2″-*O*-rhamnoside; 6. Isovitexin-2″-*O*-rhamnoside; 7. Phloretin-3′,5′-di-*C*-glucoside; 8. Diosmin; 9. Limocitrin; 10. Deoxynivalenol-3-glucuronide; 11. Swertisin-2″-*O*-rhamnoside; 12. Linariifolioside; 13. Rhoifolin; 14. Isowertin-2″-*O*-rhamnoside; 15. Linarin; 16. 1-*O*-benzoyl-17-defurano-17-(2-buten-4-olide-2-yl)salannic acid methyl ester; 17. Pyrophaeophorbide A; 18. 4*α*-formyl-4*β*-methyl-5*α*-8-cholesten-3*β*-ol. (Seed) 1. Gentiopicrin; 2. Vicenin-2; 3. 6‴-(3-hydroxy-3-methylglutaroyl)-2″-*O*-*β*-D-galactopyranosyl orientin; 4. Xanthotoxol; 5. Isovitexin-2″-*O*-rhamnoside; 6. Phloretin-3′,5′-di-*C*-glucoside; 7. Picraquassioside A; 8. Tetrahydroswertianolin; 9. Haploperoside B; 10. Swertisin-2″-*O*-rhamnoside; 11. Apigenin 7-[rhamnosyl-(1->2)-galacturonide]; 12. Isowertin-2″-*O*-rhamnoside; 13. Linarin; 14. Methoxsalen; 15. Isopimpinellin; 16. Osthenol; 17. 7-Amino-4-methylcoumarin; 18. Bergaptol; 19. Phellopterin; 20. Hexylphenazolone; 21. Homopisatin; 22. N-Methylanthranilic acid; 23. Auraptene; 24. 1-*O*-benzoyl-17-defurano-17-(2-buten-4-olide-2-yl)salannic acid methyl ester; 25. Pyrophaeophorbide A; 26. 4*α*-formyl-4*β*-methyl-5*α*-8-cholesten-3*β*-ol.

LC-MS metabolite profiling of minicitrus revealed a broad spectrum of secondary metabolite classes, including flavonoids, coumarins, and various glycosidic derivatives (Figure 2, Table 1). From the LC-MS profiles, the root contained relatively few compounds, with only four metabolites detected. The leaf, stem, and fruit exhibited more similar chemical compositions, with the leaf and stem sharing an almost identical metabolite profile. In contrast, the seed displayed a distinct chemical profile compared to the other tissues. The majority were flavone derivatives, such as *C*-glycosyl flavones (e.g., vicenin-2, orientin and their *O*-rhamnosides) and *O*-glycosyl flavones (e.g., diosmin and rhoifolin), which are among the most common phenolics in Citrus species [13]. These flavone derivatives were mainly detected in the leaf, stem, and fruit, whereas the root was characterized by coumarin-related metabolites and lacked most flavone glycosides. The seed contained several metabolites that were not detected in the other tissues, including bitter monoterpene glycosides and furanocoumarins. In addition, a limonoid-type triterpenoid, putatively annotated as a defuranated salannin analog, was detected mainly in the fruit and seed extracts.

HPLC analysis quantified 13 metabolites using authentic reference standards and semi-quantified the remaining 24 metabolites as linarin equivalents. The resulting profiles showed clear tissue-dependent differences, with the total abundance of quantified metabolites following the order leaf > stem > fruit > seed > root. Phloretin-3′,5′-di-*C*-glucoside was the predominant metabolite in the leaf, stem, and fruit, reaching 1823.19 ± 278.71, 1576.25 ± 189.18, and 847.11 ± 27.28 μg/g FW, respectively. In contrast, the root showed a highly concentrated profile dominated by xanthyletin, which reached 1760.44 ± 270.14 μg/g FW and accounted for approximately 91.5% of the total quantified metabolites in this tissue. The seed displayed a distinct quantitative profile, with 4α-formyl-4*β*-methyl-5α-8-cholesten-3*β*-ol as the most abundant metabolite and several furanocoumarins, including methoxsalen, isopimpinellin, and bergaptol, preferentially accumulated in this tissue.

### 3.2. Chemical and Cellular Antioxidant Activities of Minicitrus Extracts and Representative Compounds

**Figure 3:**
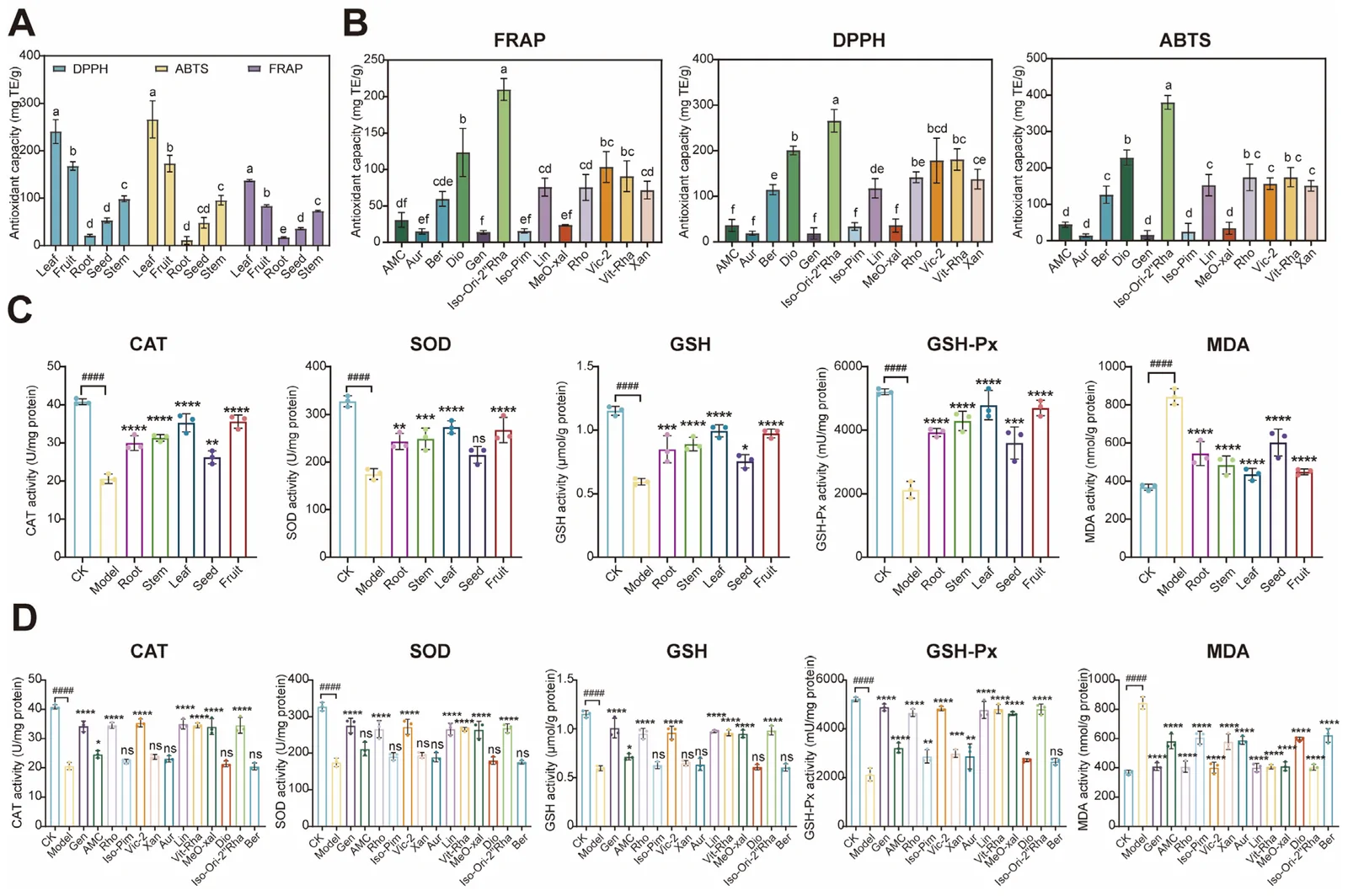
Chemical and cell-based antioxidant activities of minicitrus tissue extracts and representative standard compounds. (**A**) Chemical antioxidant capacities of extracts from five minicitrus tissues, including leaf, fruit, root, seed, and stem, as determined by DPPH, ABTS, and FRAP assays. (**B**) Chemical antioxidant capacities of 13 standard compounds determined by FRAP, DPPH, and ABTS assays. (**C**) Effects of extracts from five minicitrus tissues on cellular antioxidant-related indicators, including CAT, SOD, GSH, GSH-Px, and MDA, in the cell-based oxidative stress model. (**D**) Effects of 13 standard compounds on cellular antioxidant-related indicators, including CAT, SOD, GSH, GSH-Px, and MDA. Data are presented as mean ± SD. Statistical significance was determined by one-way ANOVA with multiple comparisons. Different lowercase letters indicate significant differences among groups in the chemical antioxidant assays (*p* < 0.05). Hash symbols indicate significant differences between the CK and model groups, whereas asterisks indicate significant differences between treatment groups and the model group. # or * *p* < 0.05, ## or ** *p* < 0.01, ### or *** *p* < 0.001, and #### or **** *p* < 0.0001; ns, not significant.

The chemical antioxidant capacities of extracts from different minicitrus tissues were evaluated using DPPH, ABTS, and FRAP assays (Figure 3A). Leaf extracts exhibited the strongest antioxidant activity across all three assays, with DPPH, ABTS, and FRAP values of 240.56 ± 25.23, 266.12 ± 39.12, and 137.17 ± 2.41 mg TE/g, respectively. These values were 43.56%, 53.71%, and 64.43% higher than those of fruit extracts, which showed the second-highest DPPH and ABTS activities. Stem extracts displayed intermediate activity, whereas seed and root extracts were substantially weaker, with root extracts showing the lowest values across the three assays. Among the 13 representative standard compounds tested (Figure 3B), Isoorientin 2″-*O*-rhamnoside exhibited the highest antioxidant capacity, reaching 265.74 ± 24.81, 379.70 ± 19.05, and 209.73 ± 15.06 mg TE/g in the DPPH, ABTS, and FRAP assays, respectively. Diosmin also showed consistently high activity, with corresponding values of 200.72 ± 9.42, 228.37 ± 20.80, and 123.25 ± 32.92 mg TE/g. Several other flavonoid-related compounds, including vicenin-2, vitexin-2″-*O*-rhamnoside, rhoifolin, and linarin, showed intermediate activities, whereas auraptene, gentiopicrin, isopimpinellin, and methoxsalen generally exhibited much lower antioxidant capacities.

**Figure A1:**
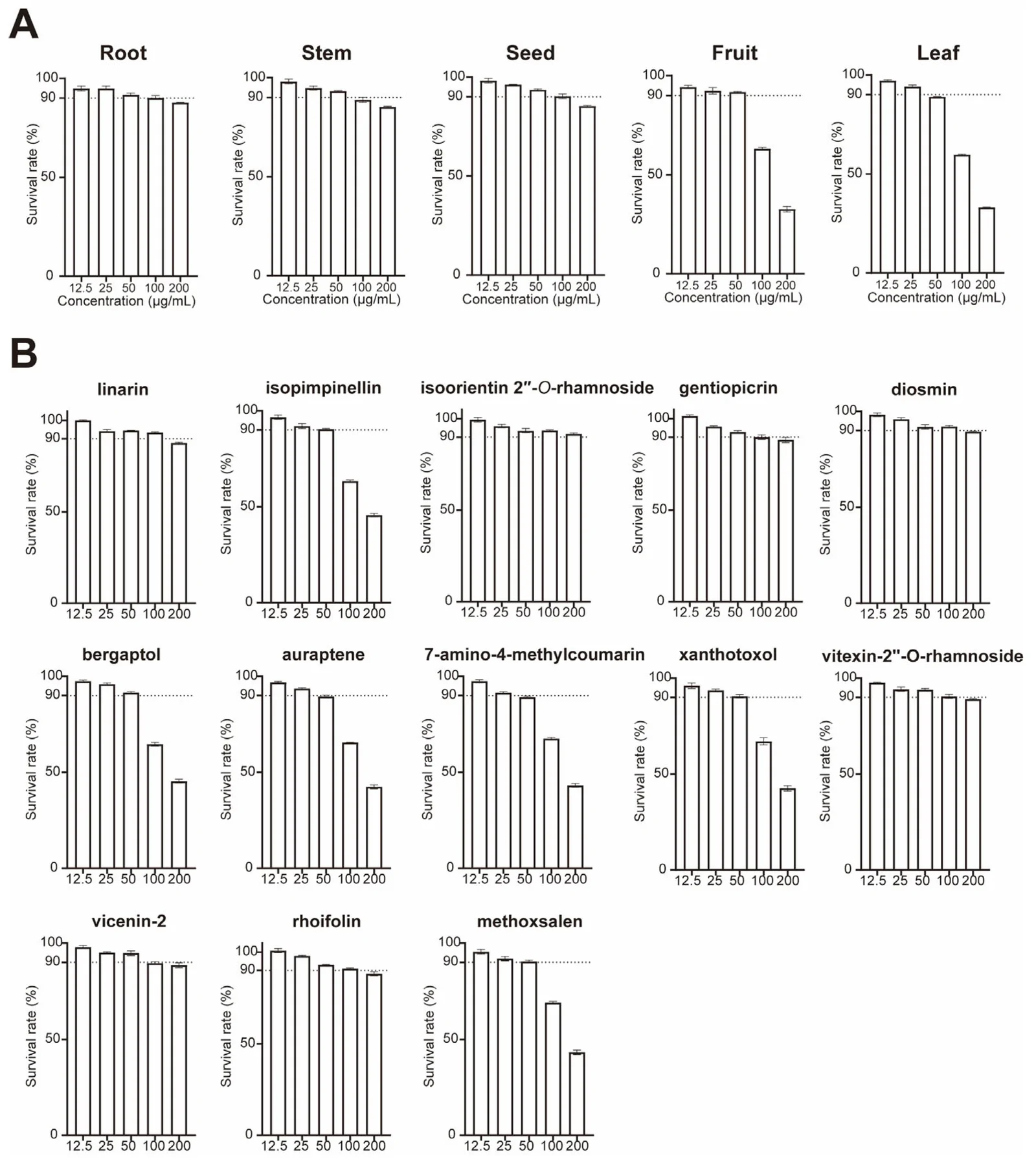
Effects of minicitrus tissue extracts and representative standard compounds on cell viability. (**A**) Effects of extracts from five minicitrus tissues, including root, stem, seed, fruit, and leaf, on cell survival rate. (**B**) Effects of 13 representative standard compounds on cell survival rate. Data are presented as mean ± SD.

Before evaluating cellular antioxidant activity, non-cytotoxic treatment concentrations were selected using the CCK-8 assay, with 90% cell viability used as the reference threshold (Figure A1). Oxidative stress markedly reduced CAT, SOD, GSH, and GSH-Px from 40.82 ± 0.75 U/mg protein, 327.50 ± 11.41 U/mg protein, 1.153 ± 0.036 μmol/g protein, and 5210.03 ± 90.47 mU/mg protein in the CK group to 20.59 ± 1.25, 174.60 ± 11.40, 0.593 ± 0.026, and 2124.27 ± 263.26, respectively, in the model group, while MDA increased from 367.73 ± 15.77 to 843.27 ± 41.67 nmol/g protein (Figure 3C). Among the tissue extracts, leaf and fruit showed the strongest overall protective effects. Relative to the model group, leaf treatment increased CAT, SOD, GSH, and GSH-Px by 71.4%, 56.4%, 67.6%, and 125.5%, respectively, and reduced MDA by 48.4%; fruit treatment produced comparable changes of 72.8%, 53.0%, 64.3%, 121.4%, and −46.8%, respectively. In contrast, the seed extract showed a weaker response, particularly for SOD, which was not significantly different from the model group. Among the 13 representative compounds tested (Figure 3D), gentiopicrin and vicenin-2 showed strong recovery across multiple cellular antioxidant indicators. Gentiopicrin increased GSH-Px activity to 4883.57 ± 140.53 mU/mg protein and reduced MDA to 408.80 ± 26.63 nmol/g protein, while vicenin-2 increased GSH-Px to 4830.07 ± 88.87 mU/mg protein and reduced MDA to 400.30 ± 37.66 nmol/g protein. Rhoifolin, linarin, vitexin-2″-*O*-rhamnoside, diosmin, and Isoorientin 2″-*O*-rhamnoside also significantly improved multiple antioxidant-related indicators, whereas isopimpinellin, auraptene, methoxsalen, and bergaptol showed weaker or non-significant effects on several endpoints.

### 3.3. Endophytic Bacterial Community Composition in Minicitrus

**Figure 4:**
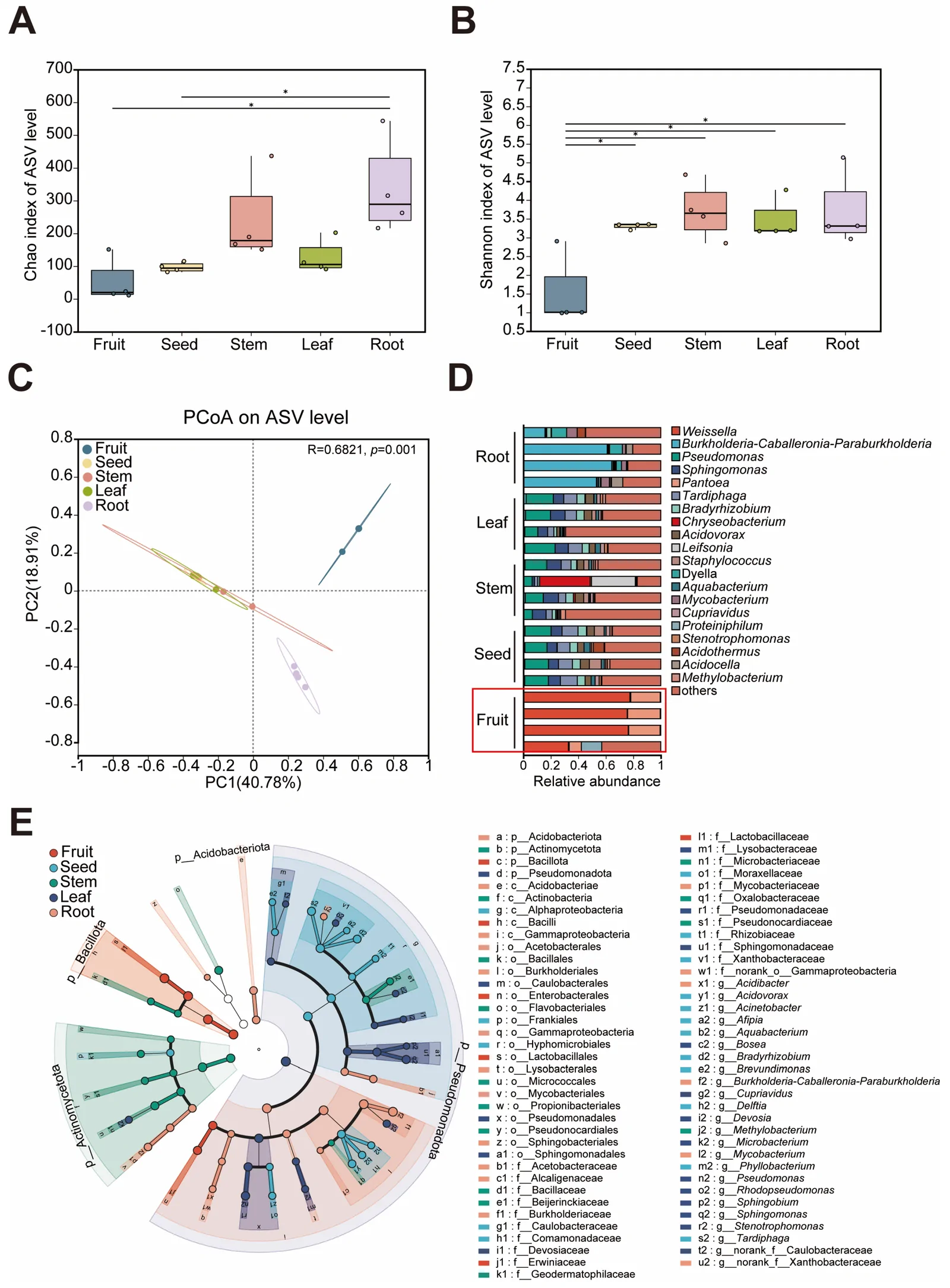
Diversity and taxonomic composition of endophytic bacterial communities in different minicitrus tissues. (**A**) Chao index of endophytic bacterial communities at the ASV level. (**B**) Shannon index of endophytic bacterial communities at the ASV level. (**C**) Principal coordinate analysis showing differences in bacterial community structure among different minicitrus tissues at the ASV level. (**D**) Relative abundance of dominant bacterial genera in different minicitrus tissues. (**E**) LEfSe cladogram showing differentially abundant bacterial taxa among minicitrus tissues from the phylum to genus levels. For panels (**A**,**B**), statistical significance was determined using the Kruskal–Wallis rank-sum test. * *p* < 0.05. For panel (**C**), group differences in bacterial community structure were tested using ANOSIM.

To explore potentially valuable endophytic bacteria in minicitrus, we profiled the endophytic bacterial communities in five tissues, including fruit, seed, stem, leaf, and root. After initial quality filtering and paired-end read merging, a total of 1,089,958 optimized sequences were retained, ranging from 40,460 to 68,459 sequences per sample. Following DADA2 denoising, dereplication, and chimera removal, 709,294 high-quality sequences remained, with 24,418–46,898 sequences per sample and an average of approximately 35,465 sequences per sample. The number of ASVs detected in individual samples ranged from 13 to 552. The raw 16S rRNA gene amplicon sequencing data generated in this study have been deposited in the NCBI under BioProject accession number PRJNA1489318. We first evaluated the alpha diversity of endophytic bacterial communities in the five minicitrus tissues. The Chao index was used to estimate bacterial richness at the ASV level (Figure 4A). Root samples exhibited the highest Chao index, whereas fruit samples showed the lowest values, indicating that roots harbored richer endophytic bacterial communities than fruit. Significant differences were observed between root and fruit, as well as between root and seed. The Shannon index was further used to evaluate bacterial alpha diversity by considering both ASV richness and community evenness (Figure 4B). Fruit samples exhibited the lowest Shannon index and were significantly different from all other tissues, indicating that the fruit-associated endophytic bacterial community had lower overall diversity. We further performed PCoA to evaluate differences in bacterial community structure among the five minicitrus tissues at the ASV level (Figure 4C). The first two principal coordinates explained 40.78% and 18.91% of the total variation, respectively. Stem, leaf, and seed samples were positioned closely, indicating relatively similar endophytic bacterial community structures among these tissues. In contrast, fruit and root samples were clearly separated from the other tissues and from each other, suggesting that they harbored more distinct endophytic bacterial communities. We next compared the taxonomic composition of endophytic bacterial communities at the genus level (Figure 4D). The top 20 most abundant genera were used to visualize the dominant bacterial groups across tissues. Consistent with the PCoA results, root and fruit samples showed distinct genus-level community profiles compared with the other tissues. The root samples were mainly dominated by the *Burkholderia-Caballeronia-Paraburkholderia* group, whereas fruit samples were characterized by high relative abundances of *Weissella* and *Pantoea*. In seed, stem, and leaf samples, *Pseudomonas* and *Sphingomonas* were among the dominant genera. LEfSe analysis was further performed to identify tissue-associated bacterial taxa at different taxonomic levels (Figure 4E). At the phylum level, fruit samples were mainly associated with Bacillota, root samples were characterized by Acidobacteriota, stem samples were associated with Actinomycetota, and leaf samples were enriched in Pseudomonadota. At the genus level, root samples were characterized by the *Burkholderia-Caballeronia-Paraburkholderia* group, *Acidibacter*, and *Mycobacterium*, while leaf samples were associated with genera such as *Pseudomonas*, *Sphingomonas*, *Sphingobium*, and *Rhodopseudomonas*. Seed samples showed enrichment of several genera, including *Acinetobacter*, *Acidovorax*, *Bradyrhizobium*, *Brevundimonas*, and *Tardiphaga*, whereas stem samples were mainly associated with *Methylobacterium*. In fruit samples, the LEfSe-enriched taxa were more concentrated and were mainly related to Bacillota and its downstream lineage. Together with the genus-level composition shown in Figure 4D, these results indicate that fruit harbored a distinct and relatively focused endophytic bacterial community dominated by *Weissella* and *Pantoea*, providing candidate taxa for subsequent isolation and validation.

### 3.4. Isolation and Identification of Culturable Endophytic Bacteria from Minicitrus Fruit

To obtain culturable fruit-associated endophytic bacteria for further validation, we isolated endophytic bacteria from minicitrus fruit using different culture media. Five culturable isolates with distinct colony colors and morphologies were obtained and subjected to 16S rRNA gene amplification and sequencing. The 16S rRNA gene sequences of the two representative isolates were deposited in the NCBI GenBank database under accession numbers PZ623460 for *Weissella* sp. SJG-1 and PZ623461 for *Pantoea* sp. SJG-3. BLAST analysis against the NCBI database was used for preliminary taxonomic identification. Based on the fruit-associated bacterial community profile described above, isolates assigned to the genera *Weissella* and *Pantoea* were selected for subsequent experiments.

**Figure 5:**
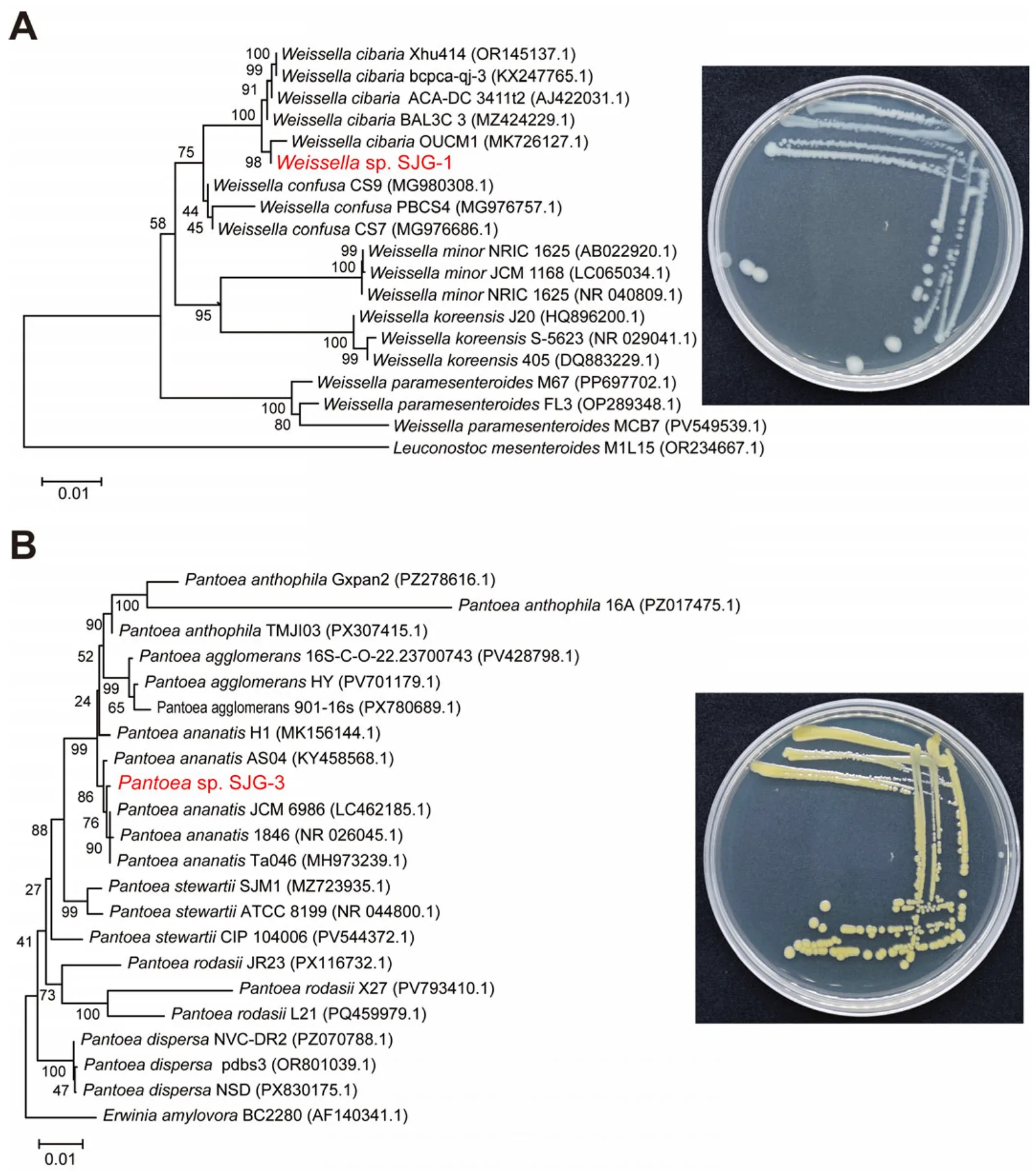
Phylogenetic identification and colony morphology of two representative culturable endophytic bacterial isolates from minicitrus fruit. (**A**) Phylogenetic tree based on 16S rRNA gene sequences showing the taxonomic placement of *Weissella* sp. SJG-1, together with representative colony morphology on agar medium. (**B**) Phylogenetic tree based on 16S rRNA gene sequences showing the taxonomic placement of *Pantoea* sp. SJG-3, together with representative colony morphology on agar medium.

Phylogenetic analysis based on 16S rRNA gene sequences further supported the taxonomic placement of the two selected isolates. SJG-1 clustered closest to *Weissella cibaria*, whereas SJG-3 clustered within the *Pantoea ananatis* clade. Given the limited species-level resolution of 16S rRNA gene analysis, the isolates were conservatively designated *Weissella* sp. SJG-1 and *Pantoea* sp. SJG-3. On agar medium, SJG-1 formed white to milky-white colonies with a round shape, smooth surface, and relatively regular margins, which was consistent with the colony morphology commonly observed for *Weissella* strains (Figure 5A). *Pantoea* sp. SJG-3 clustered within the *P. ananatis* clade, indicating a close phylogenetic relationship with *P. ananatis*. This isolate produced yellow, round, smooth colonies on agar medium, consistent with the typical yellow-pigmented colony morphology reported for *Pantoea* species, especially *P. ananatis* (Figure 5B). Based on the 16S rRNA gene phylogeny and colony characteristics, *Weissella* sp. SJG-1 and *Pantoea* sp. SJG-3 were selected as representative culturable fruit-associated endophytic isolates for downstream functional assessment.

### 3.5. Weissella sp. SJG-1 Treatment Enhances Metabolites and Antioxidant Activity in Minicitrus Fruit

**Figure 6:**
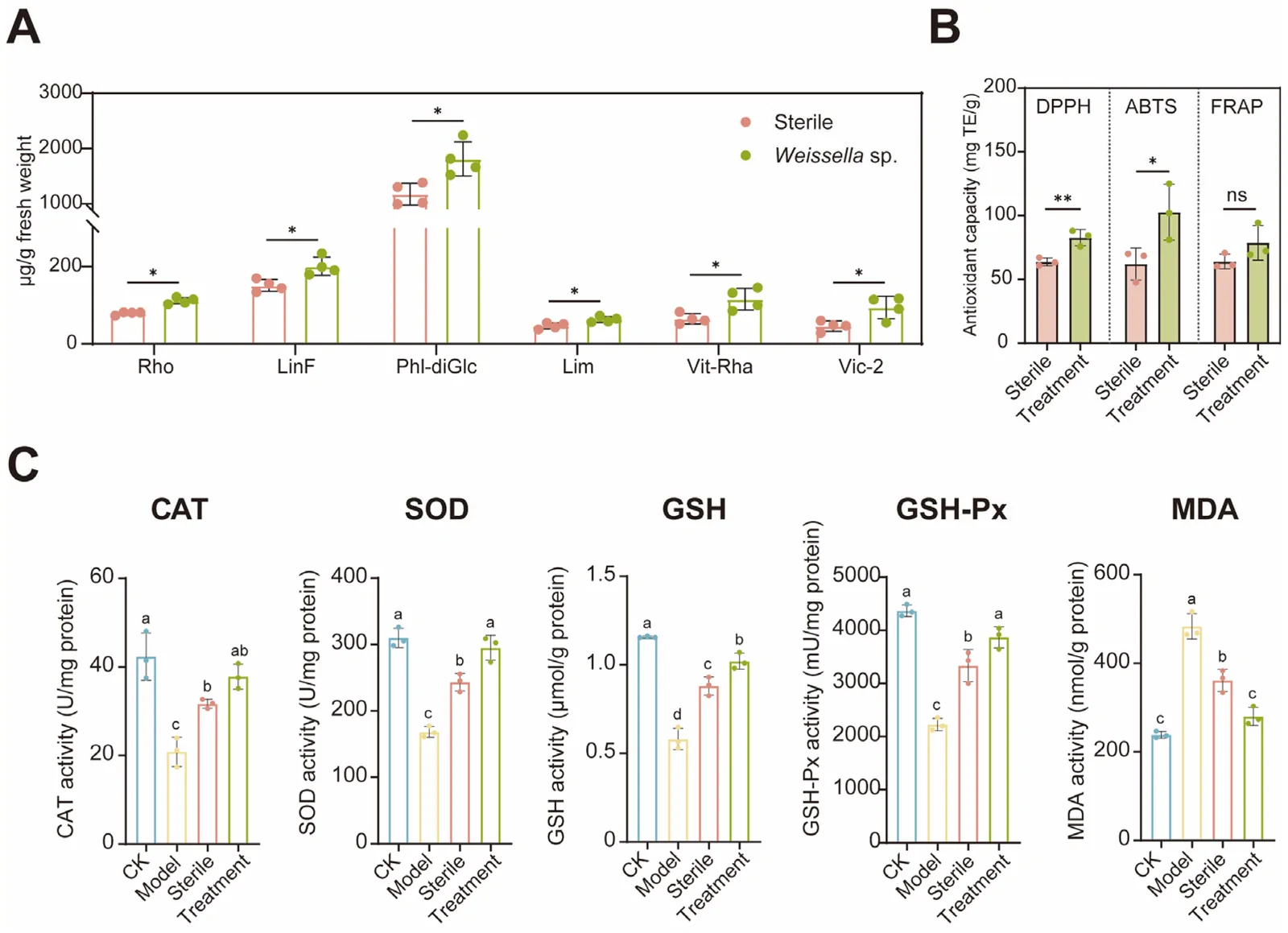
Effects of *Weissella* sp. SJG-1 treatment on metabolites and antioxidant activity of minicitrus fruit peel extracts. (**A**) HPLC-quantified metabolites significantly increased after *Weissella* sp. SJG-1 treatment (*n* = 4). (**B**) Chemical antioxidant activities of sterile and *Weissella* sp. SJG-1-treated fruit peel extracts measured by DPPH, ABTS, and FRAP assays (*n* = 4). (**C**) Cellular antioxidant-related indicators of sterile and *Weissella* sp. SJG-1-treated fruit peel extracts, including CAT, SOD, GSH, GSH-Px, and MDA (*n* = 3). Data are presented as mean ± SD. Statistical significance was determined using Student’s *t*-test for panels (**A**,**B**) and one-way ANOVA for panel C. * *p* < 0.05, ** *p* < 0.01; ns, not significant.

To evaluate whether the selected fruit-associated endophytic bacteria could modulate bioactive metabolites in minicitrus fruit, mature fruits were inoculated with *Weissella* sp. SJG-1 or *Pantoea* sp. SJG-3, and samples were collected after one day for HPLC analysis. *Pantoea* sp. SJG-3 treatment did not cause marked changes in the detected metabolites (Table A1), whereas *Weissella* sp. SJG-1 significantly increased six flavonoid-related metabolites (Figure 6A). The largest increases were observed for vicenin-2, vitexin-2″-*O*-rhamnoside, and phloretin-3′,5′-di-*C*-glucoside, which increased by 104.45%, 77.79%, and 54.06%, respectively, compared with the sterile control. Rhoifolin, linariifolioside, and limocitrin were also significantly increased following SJG-1 treatment.

These metabolite changes were accompanied by enhanced antioxidant activity of the fruit peel extracts. Compared with the sterile control, SJG-1 treatment increased DPPH and ABTS antioxidant capacities by 29.83% and 65.59%, respectively, whereas FRAP increased by 22.92% but the difference was not significant (Figure 6B). In the cellular assays, SJG-1-treated extracts increased SOD, GSH, and GSH-Px by 21.23%, 15.91%, and 15.94%, respectively, and reduced MDA accumulation by 22.51% relative to the sterile control (Figure 6C). The resulting SOD, GSH-Px, and MDA values were restored to levels comparable to those of the CK group, whereas GSH remained significantly lower than the CK level. CAT activity was 19.42% higher after SJG-1 treatment, but this difference was not statistically significant. Together, these results show that *Weissella* sp. SJG-1 treatment was associated with increased levels of selected flavonoid-related metabolites and improved chemical and cellular antioxidant properties of minicitrus fruit peel extracts.

## 4. Discussion

Citrus fruits and related small citrus species are rich sources of specialized metabolites, including flavonoids, coumarins, limonoids, and other phenolic derivatives, many of which contribute to plant defense and human health-related bioactivities [19]. In the present study, minicitrus exhibited a clear tissue-dependent distribution of phytochemicals. Leaves, stems, and fruits shared relatively similar metabolite profiles and accumulated abundant flavone glycosides, particularly *C*-glycosyl flavones and related derivatives. Among these metabolites, phloretin-3′,5′-di-*C*-glucoside was particularly abundant, reaching 1823.19 ± 278.71 μg/g FW in leaves, 1576.25 ± 189.18 μg/g FW in stems, and 847.11 ± 27.28 μg/g FW in fruits. Vicenin-2, Isoorientin 2″-*O*-rhamnoside derivatives, and vitexin or isovitexin derivatives were also prominent components of these tissues. Similar *C*-glycosyl flavones have been reported in kumquat-related germplasm, whereas common cultivated citrus fruits, such as sweet orange and mandarin, are more typically characterized by abundant flavanone glycosides, including hesperidin, narirutin, and naringin [20,21,22]. The relatively high accumulation of phloretin-3′,5′-di-*C*-glucoside in minicitrus therefore further supports a phytochemical pattern distinct from that commonly described for cultivated citrus. This profile may reflect both the genetic background of minicitrus and its close relationship with kumquat-type citrus resources. Collectively, the prominent accumulation of phloretin derivatives and several less commonly reported flavone glycosides suggests that minicitrus may represent a valuable source of structurally distinctive phenolic compounds. Biologically, this organ-specific allocation may reflect different functional requirements among vegetative, belowground, and reproductive tissues rather than simply differences in total metabolic activity. However, tissue identity is not the only factor that can shape specialized metabolite accumulation; developmental stage, genotype, environmental conditions, and source–sink relationships may also contribute to the observed profiles. Because the present study examined a single minicitrus material under one sampling condition, these tissue-associated patterns should not yet be interpreted as fixed chemotaxonomic characteristics of the species.

The strong enrichment of flavonoid glycosides in leaves and stems may be related to the physiological requirements of photosynthetic tissues. Flavonoids can contribute to ultraviolet screening, reactive oxygen species scavenging, and adaptation to environmental stress, which may explain their preferential accumulation in aerial organs exposed to light and oxidative pressure [23,24]. In contrast, the root displayed a much simpler but highly distinct profile, dominated by xanthyletin and other coumarin-related metabolites. Root-enriched coumarins have been implicated in belowground defense, interactions with soil microorganisms, and responses to biotic or abiotic stress [25]. Thus, the root profile of minicitrus may reflect a specialized chemical strategy distinct from that of aboveground tissues. The seed also showed a clearly differentiated metabolite composition, with the accumulation of furanocoumarins, bitter glycosidic compounds, and a limonoid-like triterpenoid. Citrus seeds are known to contain limonoids and other bitter or defensive compounds, which may contribute to protection against herbivory and microbial attack [26,27]. The detection of methoxsalen, isopimpinellin, bergaptol, and related compounds mainly in seeds further supports the idea that reproductive tissues of minicitrus maintain a chemically specialized defense system [28,29]. Together, these tissue-related patterns indicate that minicitrus allocates different classes of specialized metabolites to different organs, with aerial tissues mainly accumulating flavone glycosides, roots enriched in coumarin-related metabolites, and seeds maintaining a chemically specialized defense profile.

At the extract level, the leaf extract exhibited the strongest chemical and cellular antioxidant activities, followed by the fruit extract. Compared with the fruit extract, the leaf extract showed 43.56%, 53.71%, and 64.43% higher DPPH, ABTS, and FRAP antioxidant capacities, respectively, highlighting the particularly strong antioxidant potential of minicitrus leaves. This pattern is consistent with the distribution of antioxidant phytochemicals in citrus tissues, where metabolically active organs can accumulate high levels of flavonoids, phenolics, pigments, and other redox-active metabolites. In Phlegrean mandarin, peel and seed fractions contained higher levels of total flavonoids, polyphenols, ascorbic acid, and antioxidant capacity than pulp, and citrus peel has also been reported as a phenolic-rich fraction with stronger antioxidant potential than edible pulp in commercial cultivars [5,30]. The strong antioxidant activity of minicitrus leaves is consistent with their high accumulation of quantified metabolites, particularly *C*-glycosyl flavones and related derivatives. Notably, fruit extracts still displayed relatively strong antioxidant activity despite their lower total quantified metabolite abundance compared with leaves and stems, suggesting that antioxidant performance cannot be explained solely by total metabolite content. Fruits contained abundant phloretin-3′,5′-di-*C*-glucoside, linarin, isovitexin-2″-*O*-rhamnoside, swertisin-2″-*O*-rhamnoside, and other flavone glycosides, several of which also exhibited antioxidant activity in the compound-level assays. Differences in molecular abundance, hydroxylation pattern, glycosylation, and polarity can substantially influence radical-scavenging and reducing activities, making total quantified metabolite content an incomplete predictor of extract-level antioxidant capacity [31].

At the compound level, Isoorientin 2″-*O*-rhamnoside showed the strongest chemical antioxidant capacity across DPPH, ABTS, and FRAP assays, followed by diosmin. Vicenin-2, vitexin-2″-*O*-rhamnoside, rhoifolin, linarin, xanthotoxol, and bergaptol showed moderate chemical antioxidant activities, whereas gentiopicrin, auraptene, isopimpinellin, 7-amino-4-methylcoumarin, and methoxsalen showed weaker activities. The cellular assays identified a broader group of active compounds, including gentiopicrin, rhoifolin, vicenin-2, linarin, vitexin-2″-*O*-rhamnoside, diosmin, and Isoorientin 2″-*O*-rhamnoside. The difference between chemical and cellular antioxidant performance is expected for phenolic and glycosidic compounds. Chemical assays mainly measure electron transfer or hydrogen atom transfer reactions in simplified reaction systems, while cellular assays integrate membrane transport, intracellular retention, metabolic stability, enzyme regulation, glutathione homeostasis, lipid peroxidation, and the oxidative status of living cells [32,33]. Single-compound results also help interpret the antioxidant differences among tissues. The seed extract showed weaker antioxidant activity than leaf and fruit extracts, and several seed-enriched compounds, including methoxsalen, isopimpinellin, auraptene, and 7-amino-4-methylcoumarin, showed relatively weak chemical antioxidant activity in this study. A similar compound-class effect has been observed in citrus fruit fractions, where the antioxidant profile of peel, pulp, and seed changes according to the dominant phenolic classes and ripening stage [5]. Single standards provide functional markers for interpreting tissue-level antioxidant differences, while whole-extract activity also includes additive and interactive effects among coexisting metabolites.

The endophytic bacterial community of minicitrus showed clear tissue compartmentalization. Root samples harbored the highest bacterial richness, while fruit samples showed the lowest Chao and Shannon indices. This pattern agrees with the general organization of plant-associated microbiomes, in which roots act as a major interface for recruiting soil-derived microorganisms and often contain richer bacterial communities than aboveground or reproductive organs [34]. Studies in Arabidopsis and rice have shown that roots assemble distinct bacterial communities from surrounding soil and that the rhizosphere and endophytic compartments are shaped by both environmental sources and host selection [35,36]. Similar compartment effects have also been reported in perennial and horticultural plants, including grapevine and citrus, where root-associated communities differ strongly from leaf- or fruit-associated microbiota [37,38]. In minicitrus, PCoA further separated root and fruit samples from the other tissues, indicating that tissue type influenced both bacterial richness and community structure. Roots may serve as a major reservoir of endophytic bacterial ASVs, whereas fruit maintained a less diverse but clearly differentiated bacterial community.

The fruit endosphere appeared to be a selective microbial niche. Although fruit samples had the lowest alpha diversity, their genus-level composition was highly concentrated, with *Weissella* and *Pantoea* accounting for 65.64% and 19.13% of the relative bacterial abundance, respectively, and together representing 84.77% of the fruit-associated bacterial community. This highly concentrated community structure was more pronounced than that reported for several other citrus tissues. Aboveground plant organs, including leaves, flowers, and fruits, are exposed to fluctuating moisture, radiation, nutrients, and host-derived antimicrobial metabolites, which can impose strong selective pressure on microbial colonization [39,40]. Fruit tissues also provide a chemically distinct environment shaped by sugars, organic acids, flavonoids, coumarins, and other specialized metabolites. These factors may restrict the number of compatible endophytes while allowing adapted taxa to become dominant. The enrichment of Bacillota-related lineages in fruit was consistent with the dominance of *Weissella*, a lactic acid bacterium commonly associated with plant-derived and fermented substrates [41]. The presence of *Pantoea* also fits its broad ecological distribution as a plant-associated genus with diverse lifestyles in leaves, fruits, seeds, and other plant tissues [42]. Thus, the low-diversity fruit microbiome should not be interpreted as biologically unimportant. Instead, the concentrated community structure suggests that minicitrus fruit may selectively harbor a limited set of bacterial taxa adapted to its internal chemical environment.

Microbiome profiling is useful for identifying candidate taxa, but amplicon data alone cannot determine whether a taxon can directly influence host metabolites or extract bioactivity. Recent plant microbiome studies have increasingly emphasized the need to move from community description to culturable isolates, synthetic communities, and inoculation-based validation [43]. Seed and endophytic bacteria can influence plant phenotypes through specific metabolites or host interactions, as shown in rice, where a seed-endophytic *Sphingomonas* strain contributed to disease resistance through anthranilic acid production [9]. In the present study, the combination of fruit relevance, low-diversity community structure, and high relative abundance of *Weissella* and *Pantoea* supported the selection of these genera for isolation and subsequent inoculation experiments. These two genera occupy different ecological positions in plant-associated microbial communities. *Weissella* belongs to lactic acid bacteria and is frequently associated with plant-derived substrates and fermentation environments, where it can adapt to carbohydrate-rich niches and contribute to acidification, polysaccharide production, and microbial interactions [41]. Its dominance in minicitrus fruit may reflect the sugar-rich and chemically selective fruit microenvironment. *Pantoea* is a highly versatile plant-associated genus with members reported from leaves, seeds, fruits, and other plant tissues, including strains with epiphytic, endophytic, pathogenic, or plant-beneficial lifestyles [42]. The coexistence of these two genera in minicitrus fruit suggests that the fruit endosphere may support both fermentative bacteria adapted to nutrient-rich fruit tissues and broadly plant-associated bacteria with flexible ecological strategies. We retained the names *Weissella* sp. SJG-1 and *Pantoea* sp. SJG-3 because 16S rRNA-based identification provides preliminary taxonomic resolution. The recovery of these isolates allowed us to examine whether treatment with representative fruit-associated bacteria was associated with changes in minicitrus peel metabolites and antioxidant activity.

The contrasting responses to the two fruit-associated isolates suggest that the metabolic effects of bacterial treatment may be strain-dependent rather than a general consequence of bacterial exposure. Although both *Weissella* sp. SJG-1 and *Pantoea* sp. SJG-3 were isolated from minicitrus fruit, only SJG-1 treatment produced a pronounced response, characterized by coordinated increases in several flavone-related metabolites together with enhanced chemical and cellular antioxidant properties. This divergence is biologically relevant because endophytic bacteria occupying the same host tissue can differ substantially in their metabolic capabilities and interactions with the host. The response associated with SJG-1 therefore points to strain-specific traits that may influence fruit phytochemistry, rather than simply reflecting the presence of an abundant fruit-associated bacterium. However, because only representative isolates of the two dominant genera were examined, these findings should not be generalized to *Weissella* or *Pantoea* at the genus level. Further comparison of multiple strains within each genus will be required to determine whether the observed phenotype is specific to SJG-1 or represents a more broadly conserved functional trait.

The present data demonstrate that *Weissella* sp. SJG-1 treatment was associated with increased levels of several flavone-related metabolites and enhanced antioxidant activity, but they do not identify the biochemical mechanism underlying these changes. Among the possible explanations, microbial biotransformation is supported by previous studies showing that lactic acid bacteria possess glycosidases, esterases, and other enzymes capable of modifying phenolic compounds and flavonoid glycosides in plant matrices [44]. This mechanism is therefore biologically plausible for SJG-1, which belongs to a lactic acid bacterial lineage, but it was not directly tested here because microbial enzyme activities, transformation intermediates, and metabolic fluxes were not measured [45]. Host metabolic elicitation represents another literature-supported hypothesis, as endophytic bacteria can activate plant defense-related pathways, including phenylpropanoid and flavonoid metabolism [43]. However, this explanation remains unverified in the present study because host gene expression and biosynthetic enzyme activities were not examined. Other possibilities, including direct microbial production of these metabolites or indirect effects caused by changes in the local physicochemical environment, are more speculative because no direct evidence for these processes was obtained. Thus, the current experimental design does not allow us to discriminate among microbial biotransformation, host metabolic elicitation, direct microbial metabolite production, or other indirect effects, and more than one process may contribute to the observed response. At the cellular level, the recovery of SOD, GSH-Px, and MDA toward CK levels further indicated an improvement in cellular antioxidant status following *Weissella* sp. SJG-1 treatment. These findings provide preliminary strain-level evidence of an association between *Weissella* sp. SJG-1 treatment and changes in the phytochemical and functional properties of minicitrus fruit. However, the persistence and internal colonization of *Weissella* sp. SJG-1 in fruit tissues were not directly verified after inoculation. Therefore, the observed changes should be interpreted as treatment-associated effects rather than unequivocal evidence that stable endophytic colonization by SJG-1 was responsible for the altered phytochemical and antioxidant properties.

The relationship between endophytic microorganisms and plant specialized metabolites is likely to be reciprocal rather than unidirectional. Endophytes are metabolically active components of plant tissues and can themselves produce diverse bioactive secondary metabolites, while some endophytic microorganisms have also been reported to produce compounds resembling those of their host plants [46]. For example, an endophytic *Rhodococcus* sp. isolated from *Ginkgo biloba* was recently shown to produce flavonoid-related metabolites in axenic fermentation, with several constituents resembling those detected in the host leaves [47]. Such direct microbial production represents one potential route by which endophytes may contribute to the chemical environment of plant tissues, although the present study does not demonstrate de novo production of the flavone glycosides detected after *Weissella* sp. SJG-1 treatment. Conversely, plant specialized metabolites can influence microbial recruitment, persistence, and community assembly [48]. In Arabidopsis, root-secreted coumarins selectively altered bacterial community composition and inhibited the proliferation of specific *Pseudomonas* strains [49]. This observation is relevant to the tissue-specific patterns observed in minicitrus, particularly the distinct coumarin-rich profile of roots and the abundant flavone glycosides in fruit, suggesting that phytochemical composition may itself contribute to the selection of tissue-associated microbial communities. Microorganisms, in turn, can modify the abundance and activity of plant bioactive compounds. In strawberry, treatment with *Bacillus* and *Paraburkholderia* increased fruit phenolics, flavonoids, anthocyanins, and antioxidant activity [50]. Similarly, *Lactobacillus plantarum* fermentation altered the phenolic profile of kiwifruit while increasing phenolic and flavonoid contents together with DPPH and ABTS radical-scavenging activities [51]. More recently, inoculation with the endophytic *Bradyrhizobium* sp. MA1 increased catechin and epicatechin accumulation in *Amana edulis*, providing direct evidence that a selected endophytic bacterium can modify host flavonoid accumulation [52]. Although fermentation systems are not equivalent to endophytic interactions, these studies collectively support the biological feasibility of microbe-associated enhancement of plant phytochemicals and antioxidant properties. In this context, the increases in several flavone glycosides and antioxidant activities following *Weissella* sp. SJG-1 treatment are consistent with a broader pattern of reciprocal interactions between plant metabolites and associated microorganisms.

Several study-wide limitations should also be considered when interpreting these findings. First, the tissue comparisons were conducted using minicitrus material collected at a single developmental stage and under a defined growth condition; therefore, the extent to which the observed metabolite and microbiome patterns are stable across genotypes, developmental stages, seasons, or environments remains unknown. Second, although HPLC provided targeted quantitative information for representative metabolites, only 13 compounds were quantified using authentic standards, while the remaining metabolites were semi-quantified, limiting direct comparisons of absolute abundance across structurally diverse compounds. Third, 16S rRNA gene sequencing resolves community composition but does not provide direct information on absolute bacterial abundance, metabolic activity, or functional genes. Finally, the antioxidant assays and short-term bacterial treatment experiments provide functional evidence under controlled experimental conditions but do not establish in vivo nutritional efficacy, long-term microbial persistence, or the precise biochemical pathway responsible for the observed changes. These limitations define the current scope of the study and provide clear priorities for future work integrating broader biological replication, absolute microbiome quantification, strain genomics, time-course metabolomics, and host molecular responses.

Overall, this study indicates that the biological value of minicitrus cannot be understood from metabolite abundance, antioxidant activity, or microbial community structure in isolation. Instead, the data support a tissue-dependent framework in which specialized metabolite composition, functional antioxidant properties, and associated endophytic bacteria form interconnected but not necessarily causal components of fruit and organ biology. The strong antioxidant potential of leaves highlights a largely underutilized tissue as a source of bioactive compounds, whereas the fruit-associated *Weissella* sp. SJG-1 provides a candidate microbial resource for further investigation of microbiome-mediated modulation of fruit phytochemistry. Importantly, the present study establishes these relationships primarily at the level of association and short-term functional validation rather than definitive mechanism. Further studies will be required to determine the generality and mechanistic basis of these interactions.

## 5. Conclusions

This study provides an integrated characterization of bioactive metabolites, antioxidant activity, and endophytic bacterial communities across different tissues of minicitrus. LC-MS and HPLC analyses revealed clear tissue-dependent phytochemical patterns, with leaves, stems, and fruits enriched in flavone glycosides, roots characterized mainly by coumarin-related metabolites, and seeds containing abundant furanocoumarins and related compounds. Leaf extracts showed the strongest antioxidant activity, while fruit extracts also exhibited considerable chemical and cellular antioxidant potential. The endophytic bacterial communities differed markedly among tissues, and the fruit community was dominated by *Weissella* and *Pantoea*. Among the two representative culturable fruit-associated isolates, treatment with *Weissella* sp. SJG-1 was associated with significant increases in several HPLC-quantified metabolites and enhanced chemical and cellular antioxidant activities of fruit peel extracts, whereas *Pantoea* sp. SJG-3 did not significantly alter the quantified metabolites. These findings highlight minicitrus as a source of bioactive metabolites and provide preliminary evidence that fruit-associated bacteria may contribute to variation in fruit phytochemical and functional properties.

## Figures and Tables

**Table 1:** Identification and quantification of phytochemicals in five tissues of minicitrus by LC-MS and HPLC.

Peak No.	TR (min)	[M + H]^+^ (*m*/*z*)	Fragment Ions (*m*/*z*)	[M − H]^−^ (*m*/*z*)	Fragment Ions (*m*/*z*)	Formula	Tentative Compounds	Compound Abbreviation	Mean Content ± Standard Deviation (μg/g FW) ^A^	References
Root										
1	28.71	229.0873	213.0590, 211.0761, 185.0610, 157.0655, 129.0698, 128.0637, 115.0553	\	\	C_14_H_12_O_3_	Xanthyletin	Xly	1760.44 ± 270.14	
2	34.03	299.1646	163.0477, 119.0500, 107.0517, 91.0558, 81.0699	\	\	C_19_H_22_O_3_	Auraptene	Aur	85.31 ± 24.99	
3	34.96	\	\	277.2172	233.1898, 147.0822	C_18_H_30_O_2_	Linolenic acid	LnA	53.89 ± 29.29	
4	37.34	429.3711	289.2191, 177.0912, 165.0908, 163.1102	\	\	C_29_H_48_O_2_	4*α*-formyl-4*β*-methyl-5*α*-8-cholesten-3*β*-ol	Chol-Formyl	24.40 ± 13.95	[14]
Fruit										
1	8.63	595.1665	577.1589, 457.1141, 379.0825, 325.0719	593.1515	473.1089, 383.0775, 353.0671	C_27_H_30_O_15_	Vicenin-2	Vic-2	93.00 ± 18.95	
2	10.12	595.1665	449.1100, 353.0670, 329.0671, 299.0561	593.1515	473.1113, 429.0850, 357.0626, 327.0528, 309.0418, 298.0501	C_27_H_30_O_15_	Isoorientin 7-*O*-rhamnoside	Iso-Ori-7Rha	33.83 ± 18.20	
3	10.34	595.1664	449.1096, 431.0986, 353.0662, 329.0667	593.1513	473.1098, 429.0838, 357.0620, 327.0519, 309.0414	C_27_H_30_O_15_	Isoorientin 2″-*O*-rhamnoside	Iso-Ori-2″Rha	122.77 ± 31.10	
4	10.72	387.1278	283.0875, 265.0727, 225.1468, 121.0289	385.1142	265.0939, 239.0563, 179.0352, 137.0245, 93.0345	C_17_H_22_O_10_	Hedyotoside	Hed	127.57 ± 20.89	[15]
5	11.40	579.1711	433.1146, 415.1036, 337.0719, 313.0717, 283.0609	577.1562	413.0889, 311.0568, 293.0464	C_27_H_30_O_14_	Vitexin-2″-*O*-rhamnoside	Vit-Rha	65.46 ± 8.90	
6	11.62	579.1714	433.1152, 415.1038, 337.0720, 313.0722, 283.0614	577.1566	457.1152, 413.0890, 311.0573, 293.0468	C_27_H_30_O_14_	Isovitexin-2″-*O*-rhamnoside	Iso-Vit-Rha	277.69 ± 22.50	
7	12.11	599.1975	563.1791, 497.1455, 461.1412, 431.1305, 395.1139	597.1821	477.1420, 387.1124, 357.0991	C_27_H_34_O_15_	Phloretin-3′,5′-di-*C*-glucoside	Phl-diGlc	847.11 ± 27.28	
8	12.31	609.1820	463.1250, 445.1145, 427.1036, 367.0828, 343.0825, 313.0719	607.1666	443.0990, 341.0666, 323.0567, 308.0334	C_28_H_32_O_15_	Diosmin	Dio	32.21 ± 1.67	
9	13.35	347.0756	332.0555, 315.0520, 301.0356, 289.0355	\	\	C_17_H_14_O_8_	Limocitrin	Lim	31.50 ± 1.20	
10	14.31	473.1644	351.1094, 207.0652, 175.0382, 147.0438	471.1507	265.0915, 223.0616, 205.0511, 223.0616	C_21_H_28_O_12_	Deoxynivalenol-3-glucuronide	DON-GlcA	47.80 ± 3.09	
11	14.55	593.1872	447.1312, 429.1196, 311.1086, 327.0877	591.1722	427.1039, 337.0725, 325.0725, 307.0622	C_28_H_32_O_14_	Swertisin-2″-*O*-rhamnoside	Swe-Rha	247.28 ± 5.59	
12	15.10	653.1718	347.0782, 127.0387	651.1571	589.1567, 549.1250, 507.1148, 345.0618, 344.0546, 330.0385	C_29_H_32_O_17_	Linariifolioside	LinF	75.83 ± 4.75	
13	15.94	579.1711	447.1308, 351.0873, 327.0875, 297.0766	577.1561	325.0721, 307.0610	C_27_H_30_O_14_	Rhoifolin	Rho	33.86 ± 3.77	
14	16.28	593.1864	447.1378, 351.0883, 327.0884, 297.0771	591.1721	325.0725, 307.0622	C_28_H_32_O_14_	Isowertin-2″-*O*-rhamnoside	Iso-Wer-Rha	337.71 ± 13.24	
15	18.53	593.1872	285.0886, 242.0587	591.1719	283.0622, 268.0386	C_28_H_32_O_14_	Linarin	Lin	390.79 ± 20.32	
16	34.14	299.1638	163.0419, 119.0489, 107.0492, 91.0541, 81.0697	\	\	C_19_H_22_O_3_	Auraptene	Aur	210.53 ± 104.41	
17	36.40	593.2757	533.2567, 505.2279, 460.2263	591.2596	515.2432, 500.2193	C_34_H_40_O_9_	1-*O*-benzoyl-17-defurano-17-(2-buten-4-olide-2-yl) salannic acid methyl ester	Sal-Bz	38.37 ± 16.20	[16]
18	37.17	535.2767	507.2759, 435.2547	\	\	C_33_H_34_N_4_O_3_	Pyrophaeophorbide A	PheoA	151.52 ± 30.92	[17]
19	37.33	429.3729	205.1242, 165.0930, 163.1131	\	\	C_29_H_48_O_2_	4*α*-formyl-4*β*-methyl-5*α*-8-cholesten-3*β*-ol	Chol-Formyl	760.79 ± 129.46	[14]
Stem										
1	8.62	595.1667	577.1557, 457.1136, 379.0819, 337.0714, 325.0713, 295.0604	593.1514	503.1215, 473.1102, 383.0779, 353.0679	C_27_H_30_O_15_	Vicenin-2	Vic-2	272.57 ± 11.47	
2	10.12	595.1666	449.1107, 431.0992, 353.0672, 329.0673, 299.0566	593.1513	473.1095, 429.0832, 327.0518, 298.0490	C_27_H_30_O_15_	Isoorientin 7-*O*-rhamnoside	Iso-Ori-7Rha	39.39 ± 23.26	
3	10.33	595.1668	449.1110, 353.0666, 329.0681, 300.0638, 299.0563	593.1514	473.1100, 429.0816, 357.0613, 327.0515, 309.0406	C_27_H_30_O_15_	Isoorientin 2″-*O*-rhamnoside	Iso-Ori-2″Rha	190.44 ± 70.49	
4	10.71	387.1437	207.0646, 123.0805, 121.0277	385.1140	265.0932, 239.0558, 179.0346, 137.0244	C_17_H_22_O_10_	Hedyotoside	Hed	335.31 ± 58.30	
5	11.40	579.1713	433.1148, 415.1043, 397.0936, 313.0723, 283.0613	577.1559	413.0876, 311.0556, 293.0459	C_27_H_30_O_14_	Vitexin-2″-*O*-rhamnoside	Vit-Rha	59.98 ± 35.47	
6	11.61	579.1712	433.1148, 337.0718, 313.0716, 283.0610	577.1559	457.1148, 413.0883, 311.0564, 293.0464	C_27_H_30_O_14_	Isovitexin-2″-*O*-rhamnoside	Iso-Vit-Rha	242.80 ± 28.11	
7	12.11	599.1979	563.1793, 461.1417, 431.1304, 395.1139, 383.1137, 353.1027	597.1825	477.1452, 417.1213, 387.1156, 357.1061	C_27_H_34_O_15_	Phloretin-3′,5′-di-*C*-glucoside	Phl-diGlc	1576.25 ± 189.18	
8	12.30	609.1821	463.1274, 445.1148, 367.0829, 343.0830, 313.0723	607.1666	443.1005, 371.0781, 341.0674, 323.0572, 308.0339	C_28_H_32_O_15_	Diosmin	Dio	78.18 ± 5.90	
9	13.34	347.0764	332.0549, 315.0511, 301.0352, 289.0342	\	\	C_17_H_14_O_8_	Limocitrin	Lim	41.05 ± 3.18	
10	14.30	473.1645	351.1055, 207.0649, 175.0383, 147.0424	471.1496	325.0934, 265.0929, 223.0616, 221.0823, 205.0510	C_21_H_28_O_12_	Deoxynivalenol-3-glucuronide	DON-GlcA	102.08 ± 27.15	
11	14.55	593.1874	447.1332, 429.1191, 411.1088, 327.0874	591.1716	427.1028, 367.0811, 325.0712, 307.0608	C_28_H_32_O_14_	Swertisin-2″-*O*-rhamnoside	Swe-Rha	504.35 ± 75.08	
12	15.10	653.1722	347.0787, 332.0536, 127.0386	651.1563	589.1565, 549.1242, 507.1137, 345.0612	C_29_H_32_O_17_	Linariifolioside	LinF	183.41 ± 19.53	
13	15.95	579.1715	447.1332, 351.0876, 327.0880, 297.0770	577.1557	325.0712, 307.0606	C_27_H_30_O_14_	Rhoifolin	Rho	94.70 ± 6.04	
14	16.27	593.1869	447.1344, 429.1203, 351.0875, 327.0878, 297.0769	591.1725	355.0825, 325.0722, 307.0621	C_28_H_32_O_14_	Isowertin-2″-*O*-rhamnoside	Iso-Wer-Rha	676.89 ± 115.79	
15	18.52	593.1872	285.0852	591.1713	283.0614, 268.0379	C_28_H_32_O_14_	Linarin	Lin	947.34 ± 220.64	
16	36.39	593.2761	533.2619, 505.2271, 460.2266	591.2607	515.2246, 500.2212	C_34_H_40_O_9_	1-*O*-benzoyl-17-defurano-17-(2-buten-4-olide-2-yl) salannic acid methyl ester	Sal-Bz	44.90 ± 16.72	[16]
17	37.16	535.2701	507.2766, 447.2213, 435.2558	\	\	C_33_H_34_N_4_O_3_	Pyrophaeophorbide A	PheoA	272.11 ± 142.63	[17]
18	37.33	429.3745	165.0901	\	\	C_29_H_48_O_2_	4*α*-formyl-4*β*-methyl-5*α*-8-cholesten-3*β*-ol	Chol-Formyl	345.40 ± 219.20	[14]
Leaf										
1	8.59	595.1658	577.1554, 457.1130, 427.1018, 409.0922	593.1518	473.1113, 383.0787, 353.0683	C_27_H_30_O_15_	Vicenin-2	Vic-2	525.84 ± 119.37	
2	10.10	595.1660	449.1106, 431.0979, 353.0661, 329.0662, 299.0554	593.1520	447.0947, 357.0623, 327.0520, 297.0413, 285.0412	C_27_H_30_O_15_	Isoorientin 7-*O*-rhamnoside	Iso-Ori-7Rha	154.62 ± 70.62	
3	10.32	595.1661	449.1110, 431.0992, 413.0884, 329.0674	593.1517	473.1100, 429.0838, 357.0622, 327.0521, 309.0417	C_27_H_30_O_15_	Isoorientin 2″-*O*-rhamnoside	Iso-Ori-2″Rha	465.11 ± 67.98	
4	10.69	387.1275	265.0724, 121.0278	385.1141	265.0932, 239.0564, 179.0352, 137.0245	C_17_H_22_O_10_	Hedyotoside	Hed	877.72 ± 144.33	
5	11.39	579.1709	433.1161, 415.1027, 397.0925, 313.0710	577.1566	413.0907, 311.0575, 293.0479	C_27_H_30_O_14_	Vitexin-2″-*O*-rhamnoside	Vit-Rha	366.04 ± 104.65	
6	11.61	579.1710	433.1174, 337.0713, 313.0719, 283.0607	577.1567	457.1155, 413.0899, 341.0681, 293.0477	C_27_H_30_O_14_	Isovitexin-2″-*O*-rhamnoside	Iso-Vit-Rha	1022.32 ± 224.81	
7	12.09	599.1972	563.1777, 461.1411, 431.1302, 395.1129	597.1828	477.1441, 417.1218, 387.1156, 357.1060	C_27_H_34_O_15_	Phloretin-3′,5′-di-*C*-glucoside	Phl-diGlc	1823.19 ± 278.71	
8	12.30	609.1818	463.1255, 445.1133, 367.0821, 343.0823	607.1674	443.0994, 371.0775, 323.0574, 308.0338	C_28_H_32_O_15_	Diosmin	Dio	113.64 ± 15.54	
9	13.35	347.0761	332.0553, 301.0348	\	\	C_17_H_14_O_8_	Limocitrin	Lim	151.58 ± 17.46	
10	14.31	473.1644	351.1082, 207.0655, 175.0391	471.1503	265.0931, 223.0615, 205.0509, 164.0481	C_21_H_28_O_12_	Deoxynivalenol-3-glucuronide	DON-GlcA	162.79 ± 38.02	
11	14.55	593.1868	447.1349, 429.1182, 411.1074, 327.0873	591.1722	471.1304, 427.1046, 367.0831, 325.0724, 307.0621	C_28_H_32_O_14_	Swertisin-2″-*O*-rhamnoside	Swe-Rha	794.47 ± 78.76	
12	15.11	653.1713	347.0785, 127.0385	651.1570	549.1260, 507.1160, 345.0628, 330.0394	C_29_H_32_O_17_	Linariifolioside	LinF	407.31 ± 54.58	
13	15.95	570.1710	447.1342, 429.1208, 351.0878, 327.0889, 297.0771	577.1565	307.0617, 89.0245, 59.0143	C_27_H_30_O_14_	Rhoifolin	Rho	181.87 ± 40.94	
14	16.27	593.1867	429.1219, 351.0874, 327.0888,297.0772	591.1723	355.0832, 325.0727, 307.0625, 59.0146	C_28_H_32_O_14_	Isowertin-2″-*O*-rhamnoside	Iso-Wer-Rha	919.31 ± 84.86	
15	18.53	593.1864	285.0905, 270.0526	591.1717	283.0623, 268.0378	C_28_H_32_O_14_	Linarin	Lin	1408.96 ± 177.68	
16	36.39	593.2758	533.2588, 505.2263, 460.2263	591.2600	515.2451, 500.2210	C_34_H_40_O_9_	1-*O*-benzoyl-17-defurano-17-(2-buten-4-olide-2-yl) salannic acid methyl ester	Sal-Bz	69.31 ± 28.09	[16]
17	37.16	535.2698	507.2762, 447.2208, 435.2551	\	\	C_33_H_34_N_4_O_3_	Pyrophaeophorbide A	PheoA	298.86 ± 101.45	[17]
18	37.33	429.3715	165.0917	\	\	C_29_H_48_O_2_	4*α*-formyl-4*β*-methyl-5*α*-8-cholesten-3*β*-ol	Chol-Formyl	427.09 ± 74.77	[14]
Seed										
1	7.80	357.2158	177.0539, 145.0276, 117.0324	355.1035	175.0399, 160.0165, 134.0376, 132.0212	C_16_H_20_O_9_	Gentiopicrin	Gen	243.23 ± 7.10	
2	8.63	595.1665	577.1542, 457.1125, 409.0920, 379.0815, 325.0718, 337.0713	593.1515	503.1193, 473.1101, 383.0769, 353.0669, 325.0713, 297.0773	C_27_H_30_O_15_	Vicenin-2	Vic-2	24.67 ± 4.55	
3	9.42	755.2043	449.1091, 287.0558, 127.0385	753.1896	651.1596, 591.1370, 447.0950, 285.0418	C_33_H_38_O_20_	6‴-(3-hydroxy-3-methylglutaroyl)-2″-*O*-*β*-D-galactopyranosyl orientin	Ori-Glc-GluA	83.88 ± 28.41	[18]
4	11.05	203.0344	175.0406, 147.0448, 91.0543	201.0200	173.0245, 145.0281, 117.0338	C_11_H_6_O_4_	Xanthotoxol	Xan	142.29 ± 54.92	
5	11.60	579.1714	433.1166, 415.1041, 337.0717, 313.0720, 283.0612	577.1562	457.1144, 413.0888, 293.0466	C_27_H_30_O_14_	Isovitexin-2″-*O*-rhamnoside	Iso-Vit-Rha	252.69 ± 93.41	
6	12.09	599.1977	563.1774, 461.1418, 431.1304, 395.1134	597.1828	477.1417, 417.1199, 387.1123, 357.1017	C_27_H_34_O_15_	Phloretin-3′,5′-di-*C*-glucoside	Phl-diGlc	153.11 ± 32.14	
7	12.52	\	\	397.1139	235.0618, 217.0507, 191.0713, 176.0480	C_18_H_22_O_10_	Picraquassioside A	Pic-A	45.81 ± 3.76	
8	13.96	\	\	439.1242	235.0618, 191.0716, 176.0481	C_20_H_24_O_11_	Tetrahydroswertianolin	THSwer	85.90 ± 9.43	
9	14.20	\	\	541.1561	397.1136, 235.0615, 176.0477	C_24_H_30_O_14_	Haploperoside B	Hap-B	102.01 ± 45.40	
10	14.50	593.1871	447.1305, 429.1195, 411.1086, 327.0874	591.1713	427.1035. 337.0722. 325.0721. 307.0620	C_28_H_32_O_14_	Swertisin-2″-*O*-rhamnoside	Swe-Rha	45.33 ± 31.55	
11	14.78	593.1509	287.0561, 127.0385	591.1355	529.1365, 489.1047, 447.0943, 285.0416, 255.0311	C_27_H_28_O_15_	Apigenin 7-[rhamnosyl-(1->2)-galacturonide]	Api-Rha-GalA	21.45 ± 6.57	
12	16.27	593.1870	447.1332, 429.1222, 351.0888, 327.0887, 297.0778	591.1716	367.0840, 355.0826, 337.0712, 307.0620	C_28_H_32_O_14_	Isowertin-2″-*O*-rhamnoside	Iso-Wer-Rha	77.33 ± 30.42	
13	18.52	593.1870	285.0786	591.1717	283.0621, 268.0374	C_28_H_32_O_14_	Linarin	Lin	67.29 ± 15.25	
14	21.41	217.0503	202.0315, 189.0547, 185.0236, 174.0358, 161.0621, 146.0363, 118.0417	\	\	C_12_H_8_O_4_	Methoxsalen	MeO-xal	400.63 ± 24.66	
15	24.05	247.0610	217.0239, 189.0217, 161.0250, 133.0283	\	\	C_13_H_10_O_5_	Isopimpinellin	Iso-Pim	270.25 ± 57.15	
16	28.40	231.1023	175.0396, 147.0440, 119.0490, 91.0542	229.0879	174.0322. 145.0290. 130.0422	C_14_H_14_O_3_	Osthenol	Ost	49.36 ± 8.18	
17	29.46	176.0611	134.0610, 116.0500, 106.0657	174.0564	159.0306, 131.0365	C_10_H_9_NO_2_	7-Amino-4-methylcoumarin	AMC	41.34 ± 34.55	
18	30.09	203.0345	175.0419, 147.0484, 131.0508, 129.0344, 91.0550, 65.0390	\	\	C_11_H_6_O_4_	Bergaptol	Ber	262.31 ± 79.34	
19	30.70	301.1077	233.0483, 230.0200, 218.0235, 217.0139	\	\	C_17_H_16_O_5_	Phellopterin	Phe	48.47 ± 5.57	
20	30.85	258.1499	202.0944, 172.0799, 144.0841	\	\	C_16_H_19_NO_2_	Hexylphenazolone	Hex-Phen	34.14 ± 7.70	
21	31.07	301.1077	233.0567, 218.0257, 217.0140, 173.0279, 162.0319, 134.0368	\	\	C_17_H_16_O_5_	Homopisatin	Hom	100.56 ± 14.16	
22	33.34	152.0701	134.0597, 116.0490, 106.0646, 77.0385	\	\	C_8_H_9_NO_2_	N-Methylanthranilic acid	N-Me-ANA	6.11 ± 4.82	
23	34.03	299.1647	163.0442, 119.0492, 107.0495, 91.0543	\	\	C_19_H_22_O_3_	Auraptene	Aur	77.60 ± 13.81	
24	36.41	593.2763	533.2561, 460.2255	591.2591	515.2440, 500.2209, 487.2488	C_34_H_40_O_9_	1-*O*-benzoyl-17-defurano-17-(2-buten-4-olide-2-yl) salannic acid methyl ester	Sal-Bz	8.60 ± 2.84	[16]
25	37.18	535.2696	507.2763	\	\	C_33_H_34_N_4_O_3_	Pyrophaeophorbide A	PheoA	35.37 ± 9.88	[17]
26	37.35	429.3717	165.0906, 163.1109, 149.0956	\	\	C_29_H_48_O_2_	4*α*-formyl-4*β*-methyl-5*α*-8-cholesten-3*β*-ol	Chol-Formyl	82.85 ± 15.50	[14]

Note: ^A^ The values were quantified by HPLC.

## Data Availability

The raw 16S rRNA gene amplicon sequencing data generated in this study have been deposited in NCBI under BioProject accession number PRJNA1489318. The data supporting the findings of this study are included in the article.

## Appendix A

**Table A1:** Content of individual substances in the fruits of minicitrus after treatment with the two strains.

Compound	Sterile Control (μg/g Fresh Weight)	*Weissella* sp. SJG-1 (μg/g Fresh Weight)	*Pantoea* sp. SJG-3 (μg/g Fresh Weight)
Vicenin-2	46.26 ± 13.34	94.58 ± 28.74	47.51 ± 14.73
Isoorientin 7-*O*-rhamnoside	111.73 ± 37.44	119.84 ± 25.85	56.33 ± 26.90
Isoorientin 2″-*O*-rhamnoside	71.15 ± 13.79	86.94 ± 22.06	60.28 ± 10.69
Hedyotoside	128.67 ± 27.96	145.61 ± 30.88	106.36 ± 29.85
Vitexin-2″-*O*-rhamnoside	65.11 ± 13.15	115.77 ± 27.70	49.21 ± 18.34
Isovitexin-2″-*O*-rhamnoside	179.01 ± 43.84	365.46 ± 80.39	184.14 ± 56.50
Phloretin-3′,5′-di-*C*-glucoside	1174.78 ± 197.61	1809.85 ± 311.96	1177.57 ± 174.60
Diosmin	54.34 ± 3.62	63.89 ± 11.51	52.69 ± 12.52
Limocitrin	46.83 ± 7.91	62.91 ± 7.40	52.28 ± 10.26
Deoxynivalenol-3-glucuronide	56.09 ± 20.35	112.80 ± 51.67	58.04 ± 23.48
Swertisin-2″-*O*-rhamnoside	267.66 ± 71.68	596.05 ± 235.92	240.15 ± 67.63
Linariifolioside	151.23 ± 15.39	201.01 ± 23.46	159.72 ± 19.74
Rhoifolin	79.30 ± 4.17	111.59 ± 8.29	82.66 ± 3.44
Isowertin-2″-*O*-rhamnoside	150.50 ± 14.21	201.53 ± 35.94	153.01 ± 39.45
Linarin	717.63 ± 179.23	1219.48 ± 350.03	729.97 ± 124.80
Auraptene	201.37 ± 37.83	182.79 ± 19.80	199.71 ± 24.67
1-O-benzoyl-17-defurano-17-(2-buten-4-olide-2-yl) salannic acid methyl ester	36.99 ± 5.18	38.80 ± 3.16	37.12 ± 2.92
Pyrophaeophorbide A	144.60 ± 19.20	147.50 ± 15.57	136.52 ± 19.69
4alpha-formyl-4beta-methyl-5alpha-8-cholesten-3beta-ol	699.54 ± 55.71	705.93 ± 46.98	687.64 ± 43.53
